# DNA Methylation Mediates the Association Between Individual and Neighborhood Social Disadvantage and Cardiovascular Risk Factors

**DOI:** 10.3389/fcvm.2022.848768

**Published:** 2022-05-19

**Authors:** Yi Zhe Wang, Wei Zhao, Farah Ammous, Yanyi Song, Jiacong Du, Lulu Shang, Scott M. Ratliff, Kari Moore, Kristen M. Kelly, Belinda L. Needham, Ana V. Diez Roux, Yongmei Liu, Kenneth R. Butler, Sharon L. R. Kardia, Bhramar Mukherjee, Xiang Zhou, Jennifer A. Smith

**Affiliations:** ^1^Department of Epidemiology, School of Public Health, University of Michigan, Ann Arbor, MI, United States; ^2^Department of Biostatistics, School of Public Health, University of Michigan, Ann Arbor, MI, United States; ^3^Urban Health Collaborative, Drexel University, Philadelphia, PA, United States; ^4^Department of Epidemiology and Biostatistics, Dornsife School of Public Health, Drexel University, Philadelphia, PA, United States; ^5^Division of Cardiology, Department of Medicine, Duke University School of Medicine, Durham, NC, United States; ^6^Department of Medicine, Division of Geriatrics, University of Mississippi Medical Center, Jackson, MS, United States; ^7^Survey Research Center, Institute for Social Research, University of Michigan, Ann Arbor, MI, United States

**Keywords:** social epigenomics, socioeconomic status, adversity, epigenome-wide association study, obesity, diabetes, hypertension

## Abstract

Low socioeconomic status (SES) and living in a disadvantaged neighborhood are associated with poor cardiovascular health. Multiple lines of evidence have linked DNA methylation to both cardiovascular risk factors and social disadvantage indicators. However, limited research has investigated the role of DNA methylation in mediating the associations of individual- and neighborhood-level disadvantage with multiple cardiovascular risk factors in large, multi-ethnic, population-based cohorts. We examined whether disadvantage at the individual level (childhood and adult SES) and neighborhood level (summary neighborhood SES as assessed by Census data and social environment as assessed by perceptions of aesthetic quality, safety, and social cohesion) were associated with 11 cardiovascular risk factors including measures of obesity, diabetes, lipids, and hypertension in 1,154 participants from the Multi-Ethnic Study of Atherosclerosis (MESA). For significant associations, we conducted epigenome-wide mediation analysis to identify methylation sites mediating the relationship between individual/neighborhood disadvantage and cardiovascular risk factors using the JT-Comp method that assesses sparse mediation effects under a composite null hypothesis. In models adjusting for age, sex, race/ethnicity, smoking, medication use, and genetic principal components of ancestry, epigenetic mediation was detected for the associations of adult SES with body mass index (BMI), insulin, and high-density lipoprotein cholesterol (HDL-C), as well as for the association between neighborhood socioeconomic disadvantage and HDL-C at FDR *q* < 0.05. The 410 CpG mediators identified for the SES-BMI association were enriched for CpGs associated with gene expression (expression quantitative trait methylation loci, or eQTMs), and corresponding genes were enriched in antigen processing and presentation pathways. For cardiovascular risk factors other than BMI, most of the epigenetic mediators lost significance after controlling for BMI. However, 43 methylation sites showed evidence of mediating the neighborhood socioeconomic disadvantage and HDL-C association after BMI adjustment. The identified mediators were enriched for eQTMs, and corresponding genes were enriched in inflammatory and apoptotic pathways. Our findings support the hypothesis that DNA methylation acts as a mediator between individual- and neighborhood-level disadvantage and cardiovascular risk factors, and shed light on the potential underlying epigenetic pathways. Future studies are needed to fully elucidate the biological mechanisms that link social disadvantage to poor cardiovascular health.

## Introduction

Associations of individual-level socioeconomic adversity with increased risk of cardiovascular disease (CVD) are well established. The effects of socioeconomic adversity are thought to accumulate throughout a person's lifespan, as both childhood and adulthood socioeconomic status (SES) can influence CVD and its risk factors, including obesity (1–3), hypertension (4–6), diabetes (7, 8), and dyslipidemia (9–12) at a later stage (13). There is also increasing evidence suggesting that neighborhood-level social features are associated with CVD outcomes and risk factors, independent of individual-level SES (14–18). For example, in the Multi-Ethnic Study of Atherosclerosis (MESA), better neighborhood physical environment was associated with decreased body mass index (BMI) (19), higher availability of healthy food was associated with lower rates of hypertension (20), and neighborhoods with more healthy food and physical activity resources were associated with lower incidence of type 2 diabetes (21). Finally, ongoing exposure to a range of neighborhood characteristics including favorable food stores, more physical activity resources, better walking/physical activity environment, and higher neighborhood socioeconomic status was associated with higher odds of having an ideal cardiovascular health score in MESA (22).

Previous studies have proposed that epigenetic changes, which are important regulators of gene expression (23) that do not alter the underlying DNA sequence, might be a potential mechanism linking individual- and neighborhood-level social exposures to disease risk (24–27). Epigenetic mechanisms such as DNA methylation can change throughout the life course under the influence of environmental stimuli. In fact, epigenetic variation has been linked to several indicators of social disadvantage such as low childhood SES (28–32), low adult SES (29, 32), and living in disadvantaged neighborhoods (26, 33, 34). In MESA, three measures of SES (childhood SES, adult SES, and SES trajectories from childhood to adulthood) were associated with DNA methylation levels in genes related to stress reactivity and inflammation (29). Similarly, after adjusting for individual SES, neighborhood socioeconomic disadvantage and neighborhood social environment were also associated with DNA methylation in stress- and inflammation-related genes (33).

DNA methylation has also been shown to play a critical role in shaping CVD risk. Both global DNA methylation (35–37) and specific DNA methylation changes within certain candidate genes (38–40) have been associated with CVD or CVD-related biomarkers. More recently, large-scale epigenome-wide association studies have shown that methylation levels at individual CpG sites are associated with CVD (41–44) and its risk factors including measures of adiposity (45), diabetes (46–48), blood pressure (49), and lipid traits (50).

On these grounds, we hypothesized that DNA methylation acts as a mediator of at least some of the relationship between individual- and neighborhood-level social disadvantage and CVD risk factors. To date, only a few studies have formally examined whether epigenetic markers across the genome mediate the effects of social disadvantage on CVD risk factors. For instance, in adipose tissue from participants in the New England Family Study, epigenetic markers were found to mediate the association between two measures of individual-level social disadvantage (childhood SES and a broader construct of early life social disadvantage) and BMI in adulthood (51, 52). However, the sample size in these analyses was relatively small (*N* < 150 participants). To our knowledge, no studies have conducted mediation analysis for the association between neighborhood characteristics and CVD risk factors. Therefore, epigenome-wide studies with larger sample sizes are critically needed to comprehensively examine biological pathways linking individual- and neighborhood-level social disadvantage to CVD risk factors.

To fill this gap, we conducted formal mediation analysis to further examine the relationship between individual- and neighborhood-level social disadvantage, DNA methylation, and 11 CVD risk factors in a large, multi-ethnic, population-based sample of US adults using epigenome-wide methylation data measured from purified monocytes. We examined methylation in monocytes because they and their derived cells (i.e., macrophages and foam cells) play a crucial role in inflammation and contribute significantly to atherosclerosis, a primary cause of CVD. We then characterized the functional elements near the mediating methylation sites, examined their overlap with previously identified expression quantitative trait methylation (eQTM) sites, and tested for enrichment of biological pathways to investigate the functional role of the identified loci.

## Materials and Methods

### Study Population

The Multi-Ethnic Study of Atherosclerosis (MESA) is a population-based longitudinal study designed to identify risk factors for the progression of subclinical CVD (53). Between July 2000 and August 2002, a total of 6,814 non-Hispanic white, African American, Hispanic, and Chinese-American participants between the ages of 45 and 84 years were recruited from six study sites in the US, including Forsyth County, NC; Northern Manhattan and the Bronx, NY; Baltimore City and Baltimore Country, MD; St. Paul, MN; Chicago, IL; and Los Angeles County, CA. Each field center recruited participants from locally available sources, including lists of residents, lists of dwellings, and telephone exchanges. Between April 2010 and February 2012 (during MESA Exam 5), DNA methylation was measured in a random subsample of 1,264 non-Hispanic White, African American, and Hispanic MESA participants aged 55–94 years from four of the six study centers (Baltimore, MD; Forsyth County, NC; New York, NY; and St. Paul, MN). After excluding respondents with missing data on one or more variables, we focused on a subset of 1,154 individuals that had exposure, covariate and DNA methylation data for our final analyses. This study was approved by the Institutional Review Boards of all MESA field centers, the MESA Coordinating Center, and the University of Michigan.

### Measures

#### DNA Methylation

Detailed description of DNA methylation profiling and data processing procedures can be found in Liu et al. (54). Briefly, blood was drawn in the morning after a 12 h fast. Monocytes were purified using Auto-MACs automated magnetic separation units (Miltenyi Biotec, Bergisch Gladbach, Germany). A random assignment technique was used to plate the samples in order to mitigate bias due to batch, chip, and position effects. Methylation profile was measured using the Illumina HumanMethylation450 Beadchip. Bead-level methylation data were summarized using Illumina GenomeStudio. The *lumi* package for R with default settings was used for quantile normalization (55). Checks for sex and race/ethnicity mismatches and outlier identification were performed using multidimensional scaling plots. Criteria for probe elimination included: “detected” methylation levels in <90% of MESA samples (detection *p*-value cut-off = 0.05), existence of a SNP within 10 base pairs of the target CpG site, and overlap with a non-unique region as suggested by DMRcate (56). In addition, 65 probes that measured single nucleotide polymorphisms (SNPs) rather than methylation were excluded. After these eliminations, we had a total of 403,648 autosomal probes for subsequent analyses. The raw methylation level for each site was normalized and computed as an M-value, the log ratio of methylated to unmethylated signal intensity. Even though the target cell type was monocytes, there were still some residual components from other cell types in the samples. Thus, prior to analysis, each methylation site was adjusted by regressing the methylation M-value on the estimated cell proportions of residual non-monocytes (neutrophils, B cells, T cells, and natural killer cells) and adding methylation chip and position as random effects to account for any sample contamination and batch effects. Information on the nearest gene and associated genomic features of the CpG sites were from Illumina-provided annotation files (57).

#### Socioeconomic Status

Maternal educational attainment was used as an indicator of childhood SES (58). At Exam 2, respondents reported the highest level of education that their mother had completed. Available response options were no school, high school degree, some college but no degree, college degree, and graduate or professional school. We created a binary variable for maternal education (less than high school = 1; high school degree or more = 0).

Adult SES was indicated by the highest level of educational attainment of the respondent, which was reported at Exam 1. Available response options were no school, grades 1–8, grades 9–11, completed high school or GED, some college but no degree, technical school certificate, associated degree, bachelor's degree, and graduate or professional school. We created a dummy variable for respondent educational attainment (less than college = 1; college degree or more = 0).

#### Neighborhood Characteristics

Respondents' current home addresses were collected at each MESA examination, as well as during midpoint follow-up calls between exams. Based on the information collected, an address history between Exams 1 and 5 was created for each respondent and used to construct two summary scores for neighborhood exposures (see Section Supplementary Methods in Supplementary Material for additional details).

Briefly, a neighborhood socioeconomic disadvantage score was created based on the first principal component (PC) from a principal components analysis (PCA) of 16 census-tract level variables reflecting dimensions of education, occupation, income and wealth, poverty, employment, and housing from the 2000 US Census, American Community Survey 2005–2009, and American Community Survey 2007–2011. Weighted scales were created by multiplying the PC weights by the standardized variables. The first PC was highly weighted for education, occupation, and income and wealth.

For neighborhood social environment, we assessed three neighborhood dimensions collected in 2003–2005 and 2010–2012: aesthetic quality, safety, and social cohesion. Information was obtained from questionnaires administered to MESA participants and to a random auxiliary sample of other neighborhood residents in the New York, Baltimore, and Forsyth county study sites in 2003–2005 (*N* = 12,034) and all study sites in 2010–2012 (*N* = 8,553) (59). Responses of residents within the same Census tract were aggregated to create neighborhood-level measures for each of the neighborhood dimensions investigated. Conditional empirical Bayes (CEB) estimates for the three scales were constructed as a summary index, controlling for study site, respondent age and sex, and survey type (MESA or auxiliary sample). A summary social environment scale was calculated by summing the standardized CEB estimates for the three scales.

In order to assess the effects of long-term exposure to neighborhood characteristics, we used the weighted cumulative average of each of the neighborhood measures across all available MESA examinations (maximum of 5 exams, spanning 10 years from baseline to Exam 5), with the weights proportional to the number of months the respondents resided in each neighborhood. Neighborhood variables were coded so that higher values indicate greater disadvantage or less desirable social environment.

#### CVD Risk Factors and Medications

We examined a total of 11 CVD risk factors measured at Exam 5 including BMI, waist circumference (WC), systolic blood pressure (SBP), diastolic blood pressure (DBP), triglycerides (TG), high-density lipoprotein cholesterol (HDL-C), low-density lipoprotein cholesterol (LDL-C), total cholesterol (TC), serum glucose, serum insulin, and hemoglobin A1c (HbA1c). In MESA, blood samples were drawn after a 12-h fast. Measurement methods for the CVD risk factors have been described previously (60, 61). In our study, log-transformation was applied to TG, HDL-C, glucose, insulin, and HbA1c. Extreme outliers in the CVD risk factors, defined as values >3 times the interquartile range below the first quartile or above the third quartile, were removed from the analyses.

Information on current medications was collected using the medication inventory method in MESA (62, 63). Participants were asked to bring their medications to the clinic. The interviewers then transcribed the names, strengths, and dosage of the medications. Specifically, we collected information on lipid-lowering drugs, antihypertensives, and diabetes medications.

### Statistical Analysis

#### Associations Between Individual SES/Neighborhood Characteristics and CVD Risk Factors

Before formally testing for mediation, we first tested whether there was a significant association (total effect) between individual and neighborhood-level disadvantage (exposures) and CVD risk factors (outcomes). The associations between each exposure and outcome were examined separately. Model 1a tested associations of individual-level exposures (childhood SES and adult SES) with CVD risk factors, and included adjustment for age, gender, race/ethnicity, current smoking status, medication use (as appropriate), and the first 5 genetic principal components (PCs) of ancestry. Smoking was adjusted because previous studies show that many of the CpG sites associated with measures of SES are also highly associated with smoking (64). Here, we sought to identify effects of social disadvantage that were not confounded by or mediated by smoking. Genetic PCs were included because they were necessary to control for population stratification and admixture in subsequent analytic steps (i.e., when testing for mediation of the total effect by epigenetic markers, described below). We included lipid-lowering drug use as a covariate for associations with lipid traits (TC, TG, LDL-C, and HDL-C), antihypertensive use for associations with blood pressure measurements (SBP and DBP), and use of diabetes medications for associations with glycemic traits (serum glucose, insulin, and HbA1c). Model 1b tested associations of neighborhood-level exposures (neighborhood socioeconomic disadvantage and neighborhood social environment) with CVD outcomes and included all covariates in Model 1a as well as childhood SES and adult SES. *P*-values were controlled for multiple testing using Bonferroni correction (Bonferroni-corrected *p* < 0.0011, accounting for 44 tests from 11 CVD risk factors and 4 exposures).

For the significant associations in Models 1a/1b where BMI was not the dependent variable, we further adjusted for BMI (Models 2a/2b). BMI is often included in adjustment models or sensitivity analyses when studying the effect of methylation on CVD risk factors (46–50), because it may causally influence methylation at multiple loci (65). BMI may also be on the mediating pathway between social disadvantage and CVD risk factors. Thus, we wanted to explore associations between social disadvantage and CVD risk factors both before and after controlling for this potential source of confounding. Significant associations with BMI in Models 1a/1b (Bonferroni-corrected *p* < 0.0011), and significant associations with other CVD risk factors in Models 1a/1b (*p* < 0.0011) that remained associated at *p* < 0.05 after adjusting for BMI (Models 2a/2b) were selected for mediation analysis.

#### Mediation Analysis

If a significant association (total effect) was identified between an individual SES/neighborhood measure (exposure) and a CVD risk factor (outcome), we conducted epigenome-wide mediation analysis to identify specific CpG sites whose methylation may mediate at least some of the effect of the exposure on the corresponding outcome, using a cross product-based mediation approach where the mediation effect can be defined as the product of the exposure-mediator effect and the mediator-outcome effect. To obtain these two parameters for a given outcome and exposure, we used linear regression models to separately estimate the association of individual- and neighborhood-level social disadvantage with DNA methylation (the mediator) while adjusting for covariates (Equation 1), and the association of DNA methylation with the CVD risk factor while controlling for the corresponding exposure (i.e., individual or neighborhood social measure) and the same set of covariates (Equation 2). The covariates included in the regression models were the same as the covariates used in Models 1a/1b or 2a/2b to test the total effect of the exposure on the outcome. Model specification for a given outcome and exposure is provided below:


(1) $$E\left(M_{i}\right)=\;\alpha_{a}A_{i}+\;\alpha_{c}C_{i}$$

(2) $$E\left(Y_{i}\right)=\;\beta_{m}M_{i}+\;\beta_{a}A_{i}+\beta_{c}C_{i}$$

*M*_*i*_: DNA methylation (M-value) for individual *i*.

*A*_*i*_: exposure (individual SES or neighborhood characteristic) for individual *i*.

*C*_*i*_: adjustment covariates for individual *i*.

*Y*_*i*_: CVD risk factor for individual *i*.

Using the models described above, the epigenetic mediation effect can be quantified by testing:


(3) $$H_{0}:\;\alpha_{a}\beta_{m}=0$$

(4) $$H_{A}:\;\alpha_{a}\beta_{m}\neq 0$$

The null hypothesis is therefore comprised of three sub-null hypotheses: (1) α_*a*_ = 0, β_*m*_≠0; (2) α_*a*_≠0, β_*m*_ = 0; (3) α_*a*_ = 0 , β_*m*_ = 0. To test the mediation effect, we used the JT-Comp method (66) implemented in R, which takes into account the composite nature of the null hypothesis and provides well-calibrated *p*-values under the null. False discovery rate (FDR) was calculated from the mediation *p*-values to control for multiple testing. We considered a CpG site to be a significant mediator of the association between the exposure and outcome pair if it had FDR *q* < 0.05. The proportion of the total effect that was mediated by each CpG site (indirect effect) was then computed by dividing the mediation effect by the total effect.

In addition, since methylation levels may be correlated at CpG sites that are spatially close or within in the same biological pathway, we conducted PCA analysis on the identified CpG mediators to reduce their dimensionality. We extracted the first 10 PCs, capturing major axes of variability, so that we could investigate the overall effects of mediation across the identified CpG mediators. Next, the first 10 PCs for each set of CpG mediators were examined in mediation models. Specifically, for each association where significant epigenetic mediation was identified by the epigenome-wide mediation analysis, we further tested the meditation effect of each methylation PC separately using the mediate() function of the mediation R package (67). The average mediation effect and the proportion of the total effect explained by each methylation PC, as well as their corresponding confidence intervals (CIs), were obtained using non-parametric bootstrapping with 10,000 iterations.

Furthermore, to identify CpG sites that remain significant after adjusting for the other mediating CpG sites, we conducted a penalization-based multivariate high-dimensional mediation analysis on the identified CpG mediators using the HIMA package in R (68, 69). Specifically, for each association where significant epigenetic mediation was identified by the epigenome-wide mediation analysis, we fit a multivariate mediator-outcome model with all of the potential CpG mediators identified by the epigenome-wide mediation analysis and used a LASSO-based regression to estimate the effect of each CpG site on the outcome while controlling for the other CpG sites. We then obtained FDR *q*-values from the exposure-mediator model and the multivariate mediator-outcome model separately and applied the joint significance test (i.e., the maximum value among the two FDR *q*-values) to determine the significance of the mediation effect. We considered a CpG site to be a significant mediator when FDR *q* < 0.05.

As a secondary analysis, we conducted sex-stratified analysis for the association between adult SES and BMI using Model 1a and the corresponding mediation models described above. Sex-stratified analysis was warranted since effects of social factors (1, 2, 70–73) and epigenetic markers (51, 52) on BMI have been found to differ between sexes in at least some studies.

It is important to note that this mediation analysis does not necessarily establish causal pathways between social disadvantage and CVD risk factors, but rather demonstrates statistical mediation by DNA methylation using statistical criteria. Since our study has a cross-sectional design with concurrent measurement of DNA methylation and CVD risk factors, we are unable to establish temporality between the mediator and the outcome. Any CpG sites identified in the mediation analysis are therefore correlational in nature and do not necessarily infer causal mediation. Caution should be exercised in interpreting the results from the mediation analysis.

#### Genomic Feature Enrichment Analysis

Genomic feature enrichment analysis was performed on the set of significant CpG mediators for each exposure/outcome pair separately. We examined whether the genomic locations of each set of CpGs were enriched for features including gene promoters, enhancers, DNAse I hypersensitivity sites (DHS), CpG islands, and CpG island flanking shores/shelves. The genomic features and the target gene(s) from the UCSC database associated with each CpG site were obtained from the annotation files provided by Illumina (57). Specifically, we considered a CpG site to be in the promoter region if it was 0–1,500 bases upstream of a transcriptional start site. A CpG site was assigned to CpG island flanking shore/shelf if it was located within 4 kb of a CpG island. To test for enrichment, we used Fisher's exact test to compare the proportion of CpG sites that reside within a certain genomic feature among CpG mediators to the proportion of non-mediating CpG sites that reside within the same feature. For any contingency table that has a zero cell count, we added 1 to each of the cells to avoid having an odds ratio of zero.

Previously in MESA, Liu et al. identified 11,203 potential *cis*-acting regulatory CpG sites whose methylation level was associated with the expression level of any autosomal gene within 1 Mb at *q* < 0.001 (eQTMs) (54). In our study, we examined the overlap between the identified CpG mediators to these previously reported eQTMs. We also investigated whether CpG mediators were more likely to be eQTMs than non-mediating CpGs. Specifically, we performed Fisher's exact test to compare the proportion of eQTMs among our identified CpG mediators to the proportion of eQTMs among non-mediating CpG sites in our sample. Furthermore, Huan et al. identified 92 putatively causal CpG sites for cardiovascular risk factors including coronary heart disease, myocardial infarction, type 2 diabetes, SBP, DBP, HDL-C, LDL-C, TC, TG, and BMI by conducting Mendelian randomization analysis using cis-mQTLs and GWASs of the CVD risk factors (74). Since the CpG sites identified in the MR analysis using mQTLs as instrumental variables are potentially causal for cardiovascular risk factors, we examined the overlap between the identified mediating CpG sites and the 92 CpG sites reported in Huan et al. (74).

#### Gene-Set Analysis

We employed two methods to identify GO categories and KEGG pathways that may be over-represented in the identified CpG mediators. First we used the gometh() function from the missMethyl R package, which can eliminate multiple sources of bias by taking into account the number of CpG sites within a given gene and can correct for probe density bias (75). A potential limitation of this method, however, is that it maps the CpG sites to their proximal genes using chromosomal position. Yet, it is known that methylation sites do not always act on the nearest genes and might have distant regulatory effects as well. In light of this, we also conducted enrichment analysis using the enrichGO() and enrichKEGG() functions in the clusterProfiler R package (76) so that we could define the CpGs associated with gene expression using empirical data. Genes known to be associated with the CpG mediators were extracted using the methylation-gene expression pairs (eQTMs) identified previously in MESA (54), and were used as the signal gene list. All genes associated with methylation sites included in both the previous investigation and the present study were selected as the background gene list. Additionally, to ensure that any significant enrichment was not merely due to methylation sites that tend to be active in white blood cells, each CpG site was mapped to a chromatin state based on its chromosomal location using the 15-core chromatin state predicted by ChromHMM in primary mononuclear cells from peripheral blood in the Roadmap Epigenomics Project (77). Each methylation site was then classified as being in an active or inactive region depending on the predicted chromatin state. Using this information, we further restricted both the signal and background lists to only genes associated with CpG sites in an active chromatin region as a secondary analysis. GO terms and KEGG pathways with FDR *q* < 0.05 were considered significant for all gene-set analyses.

## Results

### Sample Characteristics

At Exam 5, the mean age was 69.6 years and 50.8% of the participants were female (Table 1). The sample consisted of 49.1% Non-Hispanic White, 19.4% African American, and 31.5% Hispanic participants. A total of 8.4% were current smokers, 53.4% had low childhood SES, and 66.0% had low adult SES. Neighborhood socioeconomic disadvantage ranged between −4.53 and 1.87 [mean = −0.31, standard deviation (SD) = 1.10], with a higher score indicating greater disadvantage. Neighborhood social environment ranged between −6.32 and 7.94 (mean = 0.40, SD = 2.67), with a higher score indicating a worse environment. The Pearson correlation between neighborhood socioeconomic disadvantage and neighborhood social environment was 0.26.

**Table 1:** Descriptive statistics (*N* = 1,154).

**Characteristics**	**Mean (SD) or *N* (%)**
Age (years)	69.55 (9.31)
Female	586 (50.8)
**Race/ethnicity**	
Non-Hispanic White	567 (49.1)
African-American	224 (19.4)
Hispanic	363 (31.5)
**Current smoking status**
Non-smoker	1,057 (91.6)
Smoker	97 (8.4)
**Medication use**
Antihypertensive medication	650 (56.3)
Diabetes medication (*n* = 1,142)	186 (16.3)
Lipid-lowering medication	481 (41.7)
Low childhood SES (maternal education < high school)	617 (53.4)
Low adult SES (respondent education < college)	762 (66.0)
Neighborhood socioeconomic disadvantage	−0.31 (1.10)
Neighborhood social environment	0.40 (2.67)
Body mass index (kg/m^2^, *n* = 1,152)	29.48 (5.49)
Waist circumference (cm, *n* = 1,151)	101.37 (14.02)
Systolic blood pressure (mmHg, *n* = 1,150)	123.48 (19.68)
Diastolic blood pressure (mmHg, *n* = 1,152)	68.29 (9.63)
Triglycerides (mg/dL)	110.52 (56.59)
High density lipoprotein cholesterol (HDL-C, mg/dL, *n* = 1,153)	53.95 (16.03)
Low density lipoprotein cholesterol (LDL-C, mg/dL, *n* = 1,148)	104.63 (32.45)
Total cholesterol (mg/dL)	180.43 (37.2)
Glucose (mg/dL)	103.24 (28.74)
Hemoglobin A1c (HbA1c, %, *n* = 1,148)	5.98 (0.92)
Insulin (mU/L, *n* = 1,044)	62.81 (42.12)

*SES, socioeconomic status*.

### Association Between Individual SES/Neighborhood Conditions and CVD Risk Factors

In Model 1a, low childhood SES was associated with higher SBP after Bonferroni correction (Table 2). Low adult SES was associated with higher BMI, WC, and insulin, and with lower HDL-C. In Model 1b, neighborhood socioeconomic disadvantage was associated with higher TG and with lower HDL-C. Less desirable neighborhood social environment was not associated with any CVD risk factors after correcting for multiple testing. All of the significant results from Models 1a/1b remained significant at *p* < 0.05 in Models 2a/2b (after BMI adjustment), except for the association between adult SES and WC (Table 2).

**Table 2:** Associations between individual SES/neighborhood characteristics and cardiovascular risk factors (*N* = 1,154).

**Cardiovascular risk factor**	**Childhood SES**				**Adult SES**				**Neighborhood socioeconomic disadvantage**				**Neighborhood social environment**			
	**Model 1a**		**Model 2a**		**Model 1a**		**Model 2a**		**Model 1b**		**Model 2b**		**Model 1b**		**Model 2b**	
	**β_1_**	***P*-value**	**β_1_**	***P*-value**	**β_1_**	***P*-value**	**β_1_**	***P*-value**	**β_1_**	***P*-value**	**β_1_**	***P*-value**	**β_1_**	***P*-value**	**β_1_**	***P*-value**
BMI	−0.408	0.240	n/a	n/a	**1.462**	**4.11E-05**	n/a	n/a	0.355	0.018	n/a	n/a	−0.071	0.289	n/a	n/a
WC	−0.546	0.545			**3.010**	**0.001**	−0.007	0.988	1.091	0.005			−0.129	0.463		
SBP	**4.345**	**4.26E-04**	**4.476**	**2.86E-04**	2.459	0.053			−0.301	0.575			0.141	0.555		
DBP	1.300	0.030			−0.040	0.948			−0.097	0.710			0.193	0.097		
Log (TG)	0.041	0.150			0.084	0.004			**0.048**	**1.04E-04**	**0.041**	**5.95E-04**	−0.003	0.539		
Log (HDL-C)	−0.017	0.291			–**0.055**	**0.001**	–**0.039**	**0.015**	–**0.043**	**9.95E-10**	–**0.038**	**1.37E-08**	0.004	0.257		
LDL-C	−1.428	0.451			2.641	0.177			0.137	0.868			0.382	0.304		
TC	−1.712	0.423			0.852	0.699			−1.563	0.094			0.513	0.221		
Log (Glucose)	−0.018	0.059			0.010	0.319			0.004	0.361			0.001	0.748		
Log (HbA1c)	−0.002	0.660			0.011	0.052			0.004	0.066			−0.001	0.325		
Log (Insulin)	−0.027	0.523			**0.178**	**3.24E-05**	**0.090**	**0.017**	0.026	0.152			−0.011	0.162		

*SES, socioeconomic status; BMI, body mass index; WC, waist circumference; SBP, systolic blood pressure; DBP, diastolic blood pressure; TG, triglycerides; HDL-C, high-density lipoprotein cholesterol; LDL-C, low-density lipoprotein cholesterol; TC, total cholesterol; HbA1c, hemoglobin A1c*.

*Model 1a: CVD risk factor ~ β_0_+β_1_SES+β_2_Age+β_3_Female+β_4_Black+β_5_Hispanic +β_6_Smoking+ β_7_Medication + β_8_PC1+ β_9_PC2 + β_10_PC3 + β_11_PC4 + β_12_PC5*.

*Model 1b: CVD risk factor ~ β_0_+β_1_Neighborhood+β_2_Age+β_3_Female+β_4_Black+β_5_Hispanic +β_6_Smoking+ β_7_Medication + β_8_PC1+ β_9_PC2 + β_10_PC3 + β_11_PC4 + β_12_PC5+ β_13_Childhood SES+ β_14_Adult SES*.

*Model 2a: CVD risk factor ~ β_0_+β_1_SES+β_2_Age+β_3_Female+β_4_Black+β_5_Hispanic +β_6_Smoking+ β_7_Medication + β_8_PC1+ β_9_PC2 + β_10_PC3 + β_11_PC4 + β_12_PC5+ β_13_BMI*.

*Model 2b: CVD risk factor ~ β_0_+β_1_Neighborhood+β_2_Age+β_3_Female+β_4_Black+β_5_Hispanic +β_6_Smoking+ β_7_Medication + β_8_PC1+ β_9_PC2 + β_10_PC3 + β_11_PC4 + β_12_PC5+ β_13_Childhood SES+ β_14_Adult SES+β_15_BMI*.

*Antihypertensive medication use was adjusted in models with SBP and DBP as the outcome; Lipid-lowering medication use was adjusted in models with TG, HDL-C, LDL-C, and TC as the outcome; Diabetes medication use was adjusted in models with glucose, HbA1c, and insulin as the outcome*.

*Bold text in Models 1a/1b indicates p < 0.0011 (i.e., P < 0.05 after Bonferroni correction for 44 tests). Bold text in Models 2a/2b indicates p < 0.05*.

### Mediation Analysis

Based on the above results, we investigated epigenetic mediation of the association of childhood SES with SBP, of adult SES with BMI, HDL-C, and insulin, and of neighborhood socioeconomic disadvantage with TG and HDL-C. In Model 1a, a total of 410, 5, and 7 mediating CpG sites were identified for the associations of adult SES with BMI, HDL-C, and insulin, respectively. For the significant associations where BMI was not the dependent variable, all mediating methylation sites lost significance in Model 2a after further adjustment for BMI.

Beyond the covariates in Model 1a (age, sex, race/ethnicity, current smoking status, and the first 5 genetic PCs of ancestry), adult SES explained 1.4% of the total variability in BMI. Of this relatively small total effect of adult SES on BMI, between 7.0 and 22.0% was mediated by the 410 identified CpG loci. The complete list of summary statistics and annotations of the 410 CpG sites mediating the association between adult SES and BMI in Model 1a is provided in Supplementary Table 1, and a Manhattan plot of the mediating loci is shown in Supplementary Figure 1. PCA analysis of the 410 mediating CpGs shows that the first 10 PCs cumulatively accounted for 49.5% of the variability in the methylation levels across the 410 sites (Supplementary Figure 2). After adjusting for age, sex, race/ethnicity, smoking, and the first 5 genetic PCs of ancestry, 4 of the 10 methylation PCs (PC1, PC5, PC6, and PC8) showed significant evidence of mediation at *p* < 0.05, accounting for 26.6, 4.5, 11.7, and 9.8% of the total effect of adult SES on BMI, respectively (Supplementary Table 2). The cumulative proportion of the SES-BMI association mediated by these 4 independent methylation PCs was 52.6%, suggesting that the overall mediation effect across the 410 identified CpG sites explained at least 52.6% of the association between adult SES and BMI.

When modeled jointly with a LASSO-based penalty, 5 of the 410 identified CpG sites showed significant mediation effects at FDR *q* < 0.05, explaining 0.3, 3.4, 1.2, 1.9, and 1.2% of the association between adult SES and BMI, respectively (Supplementary Table 3). In other words, 5 CpG sites showed evidence of mediating the association between adult SES and BMI even after adjusting for the other potential mediators identified in the epigenome-wide mediation analysis. Two of the identified CpG sites (cg23983783 and cg25392060) have been previously reported as eQTMs (i.e., they are associated with the expression of nearby genes). In particular, the methylation level of cg23983783 was associated with the gene expression of *N4BP2L1*, which has been associated with BMI and is involved in adipocyte homeostasis (78). Another identified CpG mediator, cg06192883 in the body of myosin 5C (*MYO5C*), encodes a protein involved in actin-based membrane trafficking. This CpG was also identified in a recent EWAS of BMI (45) and has been associated with longitudinal changes in BMI (79).

After adjusting for age, race/ethnicity, current smoking status and the first 5 genetic PCs of ancestry, adult SES explained 0.49 and 3.5% of the variability in BMI among females and males, respectively. In sex-stratified mediation analyses for BMI, we identified 12 CpG sites mediating the association between adult SES and BMI in females and males, respectively, with FDR *q* < 0.05 (Supplementary Tables 4, 5). Of the CpG mediators identified in females, all sites were also identified in the pooled analysis. In contrast, two CpG mediators (cg08448711 and cg16580391) identified in males were not statistically significant in the pooled analysis. Only one CpG site, cg15633603, was common to both sexes, which was also the site with the strongest evidence for mediation in the pooled analysis. Interestingly, CpG mediators found in females explained between 29.4 and 40.0% of the effect of adult SES on BMI, whereas those identified in males explained only 11.5–15.5%, suggesting that the epigenetic mediating pathway might be more pronounced for women.

We identified 66 mediating CpG sites for the association between neighborhood socioeconomic disadvantage and HDL-C in Model 1b. Of these, 43 methylation sites showed significant evidence of mediation even after adjusting for BMI. In Model 2b (i.e., after adjusting for age, sex, race/ethnicity, smoking, lipid-lowering drug use, the first 5 genetic PCs of ancestry, childhood SES, adult SES, and BMI), the total variability in HDL-C explained by neighborhood socioeconomic disadvantage was 2.1%, of which between 5.9 and 8.7% could be attributed to each of the 43 CpG mediators. A complete list of summary statistics and annotations of the 43 CpG sites mediating the effects of neighborhood socioeconomic disadvantage on HDL-C is shown in Supplementary Table 6, and a Manhattan plot of the mediating loci is shown in Supplementary Figure 3. As previously described, we conducted PCA on these CpG mediators to characterize the overall mediation effect across the loci. Supplementary Table 7 presents the independent mediation effects of the first 10 methylation PCs, which cumulatively accounted for 66.0% of the variability in the 43 CpG sites mediating the association between neighborhood socioeconomic disadvantage and HDL-C (Supplementary Figure 2). After controlling for the same Model 2b covariates as above, methylation PC1 and PC9 showed significant evidence of mediation (*p* < 0.05), accounting for 17.8 and 5.5% of the relationship between neighborhood socioeconomic disadvantage and HDL-C, respectively. The cumulative proportion mediated by these two uncorrelated methylation PCs was 23.3%, suggesting that the overall mediation effect across the 43 identified CpG sites explained at least 23.3% of the association between neighborhood socioeconomic disadvantage and HDL-C.

When modeled jointly with a LASSO-based penalty, 3 of the 43 identified CpG sites showed significant mediation effects at FDR *q* < 0.05 (Supplementary Table 8). The 3 identified CpG mediators each explained 1.3, 0.7, and 1.1% of the association between neighborhood socioeconomic disadvantage and HDL-C, respectively (Supplementary Table 8). In particular, cg18680181 is located in the body of *KIAA0391*, which forms an evolutionarily conserved cluster with *PSMA6* and has been reported to predispose individuals to coronary artery disease (80). In addition, haplotypes in the chromosomal region encompassing *KIAA0391* and *PSMA6* have been linked to coronary artery disease and myocardial infarction (81). Another CpG mediator, cg2475272, resides near *SETD7*, a lysine methyltransferase that methylates a wide range of targets and may play an important role in several biological processes including metabolism and immunity (82). For example, SETD7 might be involved in lipid, cholesterol, and glucose metabolism through its methylation of Farnesoid X receptor (FXR) (82).

No methylation sites showed evidence of mediation at FDR *q* < 0.05 for the associations between childhood SES and SBP, and between neighborhood socioeconomic disadvantage and TG.

### Genomic Feature Enrichment Analysis

We performed bioinformatic analysis for the 410 CpG mediators of the association between adult SES and BMI and the 43 CpG mediators of the association between neighborhood socioeconomic disadvantage and HDL-C identified using JT-Comp. The genomic feature enrichment results are shown in Table 3. Bonferroni correction accounted for testing 5 genomic features and eQTMs, described below (Bonferroni-corrected *p* < 0.0083). Compared to non-mediating methylation sites, CpG loci mediating the association between adult SES and BMI were more likely to reside within enhancer elements and CpG island flanking shores/shelves and less likely to be in CpG islands and promoter regions. Similarly, CpG loci mediating the association between neighborhood socioeconomic disadvantage and HDL-C were more likely to reside within enhancer elements and DHS, and less likely to be found in CpG islands.

**Table 3:** Genomic feature enrichment using two-sided Fisher's exact test.

**Genomic feature**	**Adult SES and BMI**		**Neighborhood socioeconomic disadvantage and HDL-C**	
	**Odds ratio**	***P*-value**	**Odds ratio**	***P*-value**
DHS	0.95	0.768	4.91	**2.03E-06**
Enhancer	2.01	**4.76E-11**	4.79	**4.84E-07**
Promoter	0.52	**5.55E-08**	0.71	0.407
Shore/shelf	1.37	**0.002**	0.62	0.197
CpG island	0.11	**1.77E-41**	0.05*	**1.07E-06**
eQTM	11.60	**1.72E-59**	11.89	**8.17E-08**

*SES, socioeconomic status; BMI, body mass index; HDL-C, high-density lipoprotein cholesterol; DHS, DNase hypersensitive site; eQTM, expression quantitative trait methylation site*.

*An odds ratio smaller than 1 indicates depletion, and an odds ratio larger than 1 indicates enrichment*.

*Bold text indicates p < 0.05 after Bonferroni correction (p < 0.0083)*.

^*^No mediating CpGs for neighborhood socioeconomic disadvantage and HDL-C were present in CpG islands. In order to conduct the enrichment analysis, we added 1 to each cell of the contingency table to avoid having an odds ratio of zero.

### Overlap With Expression Quantitative Trait Methylation and Putatively Causal CpG Sites Associated With Methylation Quantitative Trait Loci

Among the 410 CpG mediators of the association between adult SES and BMI, 93 were also previously reported eQTMs whose methylation level was associated with gene expression in a previous MESA study (54). These 93 CpG mediators were associated with the expression of 152 *cis*-genes (Supplementary Table 1). Similarly, 10 of the 43 CpG mediators of the association between neighborhood socioeconomic disadvantage and HDL-C were previously reported eQTMs associated with the expression of 14 *cis*-genes (Supplementary Table 6). Furthermore, as shown in Table 3, CpG mediators of both the associations between adult SES and BMI and between neighborhood socioeconomic disadvantage and HDL-C were highly enriched for eQTMs (Bonferroni-corrected *p* = 1.72E-59 and 8.17E-08, respectively). In other words, the identified CpG mediators were enriched for association with cis-gene expression, indicating that they were more likely to influence proximal gene expression compared to non-mediating CpG sites. None of the identified CpG mediators overlapped with the 92 putatively causal CpG sites for cardiovascular risk factors previously identified from MR analysis using cis-mQTLs as instrumental variables (74).

### Gene-Set Analysis

Mediating methylation sites were distributed across the genome (Supplementary Figures 1, 3). The 410 CpG mediators of the association between adult SES and BMI mapped to 286 unique genes. The 43 CpG mediators of the association between neighborhood socioeconomic disadvantage and HDL-C mapped to 31 unique genes. Using the Illumina annotation gene mapping, neither of the two sets of CpG mediators was enriched for any GO terms or KEGG pathways when controlling for the differing numbers of probes per gene after FDR correction. However, using the eQTM annotation from the previous MESA study (54) to map gene expression to CpG sites, the mediating CpG sites of adult SES and BMI were associated with expression of genes enriched in four GO biological process terms related to antigen processing and presentation at FDR *q* < 0.05 (Supplementary Figure 4). Similarly, mediating CpG sites of neighborhood socioeconomic disadvantage and HDL-C were associated with expression of genes enriched in nine GO terms related to biological processes such as the apoptotic process, cell death, and establishment or maintenance of cell polarity (Supplementary Figure 5). Significant GO terms for both sets of CpG mediators remained largely consistent even after excluding CpG sites located in regions of inactive chromatin in primary mononuclear cells (Supplementary Figures 6, 7). No significant over-representation in KEGG pathways was observed.

## Discussion

In this study, we assessed whether differences in DNA methylation mediate the effects of individual- and neighborhood-level social disadvantage on CVD risk factors. Epigenetic mediation was identified for associations between adult SES and/or neighborhood socioeconomic disadvantage and several CVD risk factors. The majority of these mediators lost significance after further adjustment for BMI, which indicates that BMI might also be part of the epigenetic mediation pathway for the majority of CVD risk factors. Alternatively, BMI may confound the association between social disadvantage and CVD risk factors. Our results suggest that a small fraction (1.4%) of the total variability in BMI was explained by adult SES, and that this association was partially mediated by 410 CpG sites. After reducing the CpG mediators to 10 uncorrelated PCs, we found that the set of identified CpG mediators accounted for at least 52.6% of the relationship between adult SES and BMI. Similarly, our findings show that 2.1% of the total variability in HDL-C was explained by neighborhood socioeconomic disadvantage, and this association was mediated by 43 CpG sites, independent of BMI. After reducing these 43 CpG mediators to 10 uncorrelated PCs, the overall mediation effect across the loci explained at least 23.3% of the total effect of neighborhood socioeconomic disadvantage on HDL-C. Although there was no overlap with the putatively causal CpG sites previously identified from MR analysis using cis-mQTLs as instrumental variables, the CpG mediators identified in this study could still be true statistical mediators since CpG sites influenced by social factors may be different than those influenced by genetic variants.

Many of the CpG sites reported in our study have been previously linked to CVD outcomes and risk factors. Among the 410 CpG mediators of adult SES and BMI, the top-ranking CpG sites included cg15633603 and cg10508317 in the body and 5′UTR of cytokine signaling-3 (*SOCS3*), which encodes a protein that suppresses signaling by inflammatory cytokines such as leptin and interleukin-6 signaling that are upregulated in obesity (83). Of note, one of the *SOCS3* CpGs identified in this study, cg18181703, was also identified in a recent large-scale EWAS of BMI (45), and methylation at this CpG was shown to interact with cumulative adverse life stress to influence BMI and obesity (84). Other highly ranked CpGs in our study are in the gene bodies of solute carrier 25A10 (*SLC25A10*), encoding a protein that increases lipid accumulation in adipose tissues (85), and phosphofurin acidic cluster sorting protein 2 (*PACS2*), which encodes a mitochondrial-associated membrane protein important for cellular homeostasis and implicated in obesity and other metabolic disorders including diabetes and metabolic syndrome (86).

In total, among the 410 CpG mediators of the association between adult SES and BMI, 16 sites were identified in the recent large-scale EWAS of BMI (45) (see Supplementary Table 1). Of these, two were identified as eQTMs in MESA, with cg13274938 corresponding to the C-C chemokine receptor 7 (*CCR7*), and cg10922280 corresponding to dipeptidase 2 (*DPEP2*) among other genes. The protein encoded by *CCR7* has been shown in mice to play a causal role in maintaining innate and adaptive immunity contributing to adipose tissue inflammation in obesity (87). The protein encoded by *DPEP2* has been shown to modulate macrophage inflammation (88), and is upregulated in subcutaneous white adipose tissue in obese women with type 2 diabetes (89). The most highly ranked MESA eQTM was cg19103704, which is associated with expression of the proteasome 26S subunit, ATPase 4 (*PSMC4*), a subunit of perilipin-2 which regulates intracellular lipid metabolism in macrophages, and the fc fragment of IgG binding protein (*FCGBP*), both of which have been associated with obesity (90, 91).

As a whole, we found that mediating CpG eQTMs were associated with expression of genes enriched in antigen processing and presentation, which is consistent with the well-established finding that chronic inflammation contributes to obesity and other related metabolic conditions. These inflammatory pathways remained significantly enriched even after removing CpG sites known to be in an inactive chromatin state in primary mononuclear cells from peripheral blood. The innate immune system has long been known to be implicated in the development of obesity and its associated diseases, and recent studies have also demonstrated the critical roles for cells of the adaptive immune system (92, 93). Specifically, antigen-driven responses have been linked to obesity. In prior studies, increased expression of genes involved in MHC class II antigen processing and presentation was found in adipocytes of obese women (94, 95). In mice, MHC class I antigen presentation was altered after lipid overload with dietary saturated fatty acid (96). Our results support and add to these previous studies by indicating that methylation of inflammation genes, particularly those involved in antigen processing and presentation, may mediate the effect of adult SES on BMI.

A growing body of evidence suggests that sex may influence the strength of the association between SES and obesity in developed countries (1, 2, 70–73). According to the National Health and Nutrition Examination Survey, 2005–2008, there was no significant association between education, a marker of adult SES, and prevalent obesity among men in the United States, but there was a negative relationship among women (70). In the present study, all of the CpG mediators that reached significance in sex-specific mediation analysis were exclusive to either males or females, except one. This is also consistent with findings from the New England Family Study that examined sex-specific methylation mediators between early life social disadvantage and adult BMI, where distinct CpG sites were identified for males and females (51, 52). Only two mediating CpGs identified in our combined sex analysis overlapped with those identified in the New England Family Study (cg05832823 and cg22679740), which were both identified in females only using adipose tissue. In this sample, a larger proportion of the association between SES and BMI was mediated by the CpG sites in females than males. However, in our study, females had a smaller total effect of adult SES on BMI (0.49%) than males (3.5%), which is in contrast to other studies that found stronger associations between SES and BMI in females (70). Our findings provide additional insight into the sex-specific nature of the epigenetic mechanisms linking SES to obesity and suggest that the epigenetic mediation pathways may operate more strongly for women.

A top-ranked mediator of neighborhood socioeconomic disadvantage and HDL-C, cg03128029, is located in the gene body of *NOP58*, a component of a small nucleolar ribonuclear protein (snoRNP). This CpG was previously associated with HDL in the REGICOR study (97). Interestingly, this CpG site and another top-ranking mediator of neighborhood socioeconomic disadvantage and HDL-C, cg20995564 in the zinc finger e-box binding homeobox-2 gene (*ZEB2*), were associated with serum CRP, an inflammatory biomarker of CVD, in both Europeans and African Americans (98). The two most significant eQTM mediators of neighborhood socioeconomic disadvantage on HDL-C were cg03254336, corresponding to expression of transcription factor 7-like 2 (*TCF7L2*), a major genetic risk factor for type 2 diabetes, and cg10699171, corresponding to expression of RHO family interacting cell polarization regulator 2 (*FAM65B*). Recently, it was found that dietary fat intake was more strongly associated with HDL-C in participants with a certain *TCF7L2* genetic variant (99). Genetic variants in the *FAM65B* gene region were also associated with early onset dyslipidemia, specifically hyper-LDL-cholesterolemia (100). None of the CpGs identified in our study were among the significant CpGs in the most recent EWAS of HDL-C (50).

Mediating CpG eQTMs for the association between neighborhood socioeconomic disadvantage and HDL-C were enriched for genes involved in the apoptotic process, cell death, the establishment/maintenance of cell polarity, and regulation of leukocyte chemotaxis. Lipid homeostasis is known to play a key role in regulated cell death processes including apoptosis, necroptosis, and ferroptosis (101). There is also evidence that genes playing a key role in metabolic induction of apoptosis may function through regulation of lipid metabolism, including HDL-C levels (102). Macrophage polarity, the cellular differentiation into pro-inflammatory or anti-inflammatory cells, appears to be influenced by HDL in mice but not humans (103). HDL-C is known to inhibit leukocyte chemotaxis, leading to lower levels of inflammation (104). However, more work needs to be done to fully characterize the potential pathways between genes that play a role in these cellular processes and HDL-C levels.

The methylation changes that we identified as potential mediators of the associations between social factors and CVD risk factors were in monocytes. During the development of atherosclerosis, monocytes are recruited to the endothelial cells from the bloodstream and become activated by LDL or other stimulating factors. Activated monocytes then release additional inflammatory factors such as IL-6, MCP-1, and TNF-α, and differentiate into macrophages. Finally, macrophages engulf excess lipoproteins including LDL to form foam cells, which are a key characteristic of early-stage atherosclerosis (105). Therefore, monocyte and macrophage differentiation and activation are critical processes in the development of atherosclerosis. Prior research suggests that DNA methylation plays an important role in regulating these processes. A comparison of epigenome-wide methylation among human monocytes, naive macrophages, activated macrophages with pro-inflammatory or anti-inflammatory phenotypes, and foam cells activated with oxidized or acetylated LDL found that 5,870 CpG sites were differentially methylated across the 6 cell types (106). Of these, 5,780 (98%) were attributed to monocyte-to-macrophage differentiation. In our study, 96 of the 410 (23.4%) mediating CpG sites of the association between adult SES and BMI and 23 of the 43 (53.5%) mediating CpG sites of the association between neighborhood socioeconomic disadvantage and HDL-C were also involved in monocyte-to-macrophage differentiation (Supplementary Tables 1, 6) (106). In addition, several of the mediating CpG sites were located near genes known to be implicated in the process of monocyte-to-macrophage differentiation. For example, a top-ranking mediating CpG site of the association between adult SES and BMI, cg09100014, is located in the gene body of *IRF8*, a transcription factor well-established in monocyte differentiation (107). Furthermore, as shown in Supplementary Figures 6, 7, pathway analysis using genes whose expression levels were associated with the identified CpG mediators in an active chromatin state in primary mononuclear cells showed strong enrichment for biological processes involved in monocyte-to-macrophage differentiation such as regulation of immune response, regulation of leukocyte migration, regulation of leukocyte chemotaxis.

To our knowledge, this was the first study to examine the role of DNA methylation in mediating the relationships between individual SES and neighborhood characteristics and several CVD risk factors in a large, multi-ethnic, population-based cohort. In this study, the use of composite measures for neighborhood conditions that encompass census-based and survey-based metrics allowed us to examine multiple dimensions of neighborhood. Moreover, we adopted a high-dimensional mediation method, JT-Comp, to formally test the mediation effects of hundreds of thousands CpG sites. This method takes into account the composite nature of the null hypothesis of mediation analysis, which is often ignored by other traditionally used methods such as Sobel's normality test and thus provides better calibrated *p*-values under the null (108). Other high-dimensional mediation methods including DACT (109) have been proposed more recently, but in our experience JT-Comp remains the most stable method when the mediation effects are small and sparse. Nevertheless, additional exploration with other high-dimensional mediation methods may be warranted.

Our study is not without limitations. First, DNA methylation and gene expression were measured in peripheral purified monocytes. Although blood may not be the most relevant tissue type for many of the traits we studied, it has been widely and successfully used as a surrogate for targeted tissues that are not readily accessible. For example, in a large EWAS of BMI (45), methylation at 187 CpG sites associated with BMI in blood showed a moderate to strong correlation with other metabolically relevant tissues including subcutaneous and omental fat, liver, muscle, spleen, and pancreas. Furthermore, among the 187 CpG sites associated with BMI in blood, 120 also showed directional consistency and 91 were significantly associated with BMI in adipose tissue. While these findings are consistent with the idea that methylation in blood correlates with methylation in other tissues, in another study comparing DNA methylation profile in matched samples of blood and adipose tissue, only 5.2% of the CpG sites had a >0.5 correlation between blood and adipose tissue (110). Therefore, although there is some rationale for using DNA methylation measured in blood as a proxy, association analysis in more directly relevant tissues (e.g., adipose, heart, and liver) would be the next important step to validate our results and identify additional non-blood related CpG mediators.

Second, we used education as the only indicator of individual SES. There are many other aspects of SES, such as income, occupation, and wealth, which were not examined in this study. However, education is a robust indicator of SES that correlates well with many other SES measures (111, 112). In this study, we used maternal education as a measure of childhood SES because it has been suggested as a more critical determinant of child health than paternal education (58, 113, 114). In addition to better employment and higher household income, higher maternal education might improve child health through a wide range of health-promoting behaviors and health knowledge (58). We also dichotomized both adult and parental education which may have limited our ability to detect associations with the CVD risk factors (and mediation effects). The analytical strategy we used required us to simplify analyses of individual and neighborhood social determinants to isolate their independent effects. Future work should examine joint and interaction effects and further consider the many interrelated pathways through which social determinants may impact methylation and CVD risk factors. Moreover, because analyses were adjusted for race/ethnicity, we did not investigate important ways in which structural racism may impact CVD risks and the role of methylation in these relationships. Finally, although we controlled for current smoking status as a potential confounder, residual confounding might be present because we did not take other dimensions of smoking such as intensity, duration, and pack-years into account.

In this study, we used summary neighborhood scores that were constructed as weighted averages across multiple neighborhoods in order to examine the cumulative effects of neighborhood over time. By using weights proportional to the length of time the respondents resided in each neighborhood, we assumed that neighborhoods where the respondents lived for a longer period of time would have a greater impact on the proposed mediator and outcome. However, it is possible that neighborhoods lived in more recently may have a larger influence on methylation and cardiovascular risk factors than previous neighborhoods, which was not accounted for in our analysis. Future studies should consider examining how the timing of neighborhood exposures might influence the effects of neighborhood-level social disadvantage on DNA methylation and cardiovascular risk factors. In addition, we did not have the complete address history for every respondent. Between Exams 1 and 5, 1.2 and 11.6% of respondents had at least one missing value for neighborhood SES and neighborhood social environment, respectively. This may have led to misclassification of the cumulative average neighborhood exposures for the small number of respondents affected.

Another limitation is that although we performed statistical mediation analysis, our data were cross-sectional and thus we were unable to assess the temporal relationships between changes in methylation and CVD risk factors. Thus, we cannot be fully confident of the causal direction of effect between methylation and CVD risk factors. There is some support for a causal effect of changes in methylation on BMI (79), for example. However, other studies have shown that BMI tends to be more of a driver of changes in methylation than a downstream consequence (45, 65). Furthermore, in this study, we assumed that the effect of social disadvantage on cardiovascular risk factors remains the same as the level of DNA methylation changes. Thus, we did not include exposure-mediator interaction terms in the mediation analysis. However, it is worth noting that violation of this assumption may result in invalid causal conclusions (115). Further longitudinal studies that explore exposure-mediator interaction are needed to more fully delineate the causal pathways proposed here.

Moreover, we employed an analytical strategy to first test the total effect of the exposure on the outcome, and only explored potential mediation effects when the total effect was significant. In the literature on mediation analysis, whether or not total effect should be required before testing indirect effect has been subject to considerable debate. Researchers in favor of suspending the total-effect test argue that requiring a significant total effect of the exposure on the outcome may limit the power of mediation analysis, since there are several scenarios in which the total effect may not be significant even in the presence of mediation. For example, the test of the total effect may be underpowered. Alternatively, indirect effects mediated through multiple mediators could cancel each other out, resulting in a non-significant total effect. Thus, we acknowledge that by requiring a significant total effect, our findings are conservative and may miss some true indirect effects. However, this does not invalidate our mediation analysis as we intended to assess the mediation effect, rather than the indirect effect, of DNA methylation in the relationship between social disadvantage and cardiovascular risk factors. Although the terms “mediator variables” and “indirect effects” are often used interchangeably, there is an important distinction between them (116). In particular, mediation is a special, more restrictive type of intervening relationship that implies the total effect of the exposure on the outcome was present initially and sheds light on the nature of the relationship between the exposure and the outcome [see (116) for a detailed discussion]. Therefore, given that the goal of our study was to better characterize the role of methylation in linking the effects of social disadvantage on cardiovascular diseases, we limited our search space to mediation settings with a detectable total effect.

Finally, methylation levels of CpG sites that are close to each other in the same biological pathways may be correlated. In light of this, a limitation of JT-Comp and other high-dimensional univariate mediation methods is that the correlation among potential mediators is not explicitly modeled. Recently, high-dimensional mediation methods that can jointly model potential mediators, such as the Bayesian high-dimensional mediation method (BAMA) (117) and its extensions (118, 119), have been proposed. Although these joint analysis methods can reduce the multiple testing burden, increase power, and better estimate independent effects, they are computationally heavy and can only simultaneously evaluate a few thousand mediators simultaneously. Since the goal of the current study was to conduct epigenome-wide meditation analysis with nearly half a million potential mediators, such methods were impractical, and we instead carried out *ad hoc* PCA analyses to better characterize the overall mediation effect across the identified loci and penalization-based high-dimensional mediation analysis on the potential CpG mediators identified in the epigenome-wide mediation analysis to further fine map the identified loci.

In summary, our findings support the hypothesis that DNA methylation acts as a mediator between individual- and neighborhood-level social disadvantage and CVD risk factors, and provide insight into the underlying epigenetic mechanisms that link social disadvantage to poor cardiovascular health. Since many of the identified CpG mediators are involved in the process of monocyte-to-macrophage differentiation and macrophage polarity, our findings suggest that epigenetic regulation of monocyte differentiation and subsequent activation might be a promising avenue for further investigation. Future research is needed to replicate our findings in other independent cohorts and confirm the role of DNA methylation in mediating the association between individual- and neighborhood-level social disadvantage and cardiovascular risk factors.

## Data Availability Statement

Data used in this analysis can be obtained through the MESA Data Coordinating Center (https://www.mesa-nhlbi.org/) and the database of Genotypes and Phenotypes [dbGaP accession numbers: phs000209 (MESA Cohort) and phs00420 (MESA SHARe)].

## Ethics Statement

The studies involving human participants were reviewed and approved by Institutional Review Boards (IRBs) at Johns Hopkins Medical Institutions, University of Minnesota, Columbia University Medical Center, Wake Forest University Health Sciences, University of Washington, and University of Michigan. The patients/participants provided their written informed consent to participate in this study.

## Author Contributions

YW and JS conceptualized and designed the study. BN, AD, YL, WZ, and XZ contributed to study design. AD, KM, and YL contributed to data collection, cleaning, and curation. YW conducted the data analysis, with assistance from WZ, SR, LS, JD, and YS. Result interpretation was performed by YW, JS, AD, SK, KB, XZ, BM, and YL. YW drafted the manuscript with assistance from JS, FA, and KK. JS provided financial support. All authors reviewed the results and approved the final version of the manuscript.

## Funding

MESA and the MESA SHARe project are conducted and supported by the National Heart, Lung, and Blood Institute (NHLBI) in collaboration with MESA investigators. Support for MESA is provided by Contracts 75N92020D00001, HHSN268201500003I, N01-HC-95159, 75N92020D00005, N01-HC-95160, 75N92020D00002, N01-HC-95161, 75N92020D00003, N01-HC-95162, 75N92020D00006, N01-HC-95163, 75N92020D00004, N01-HC-95164, 75N92020D00007, N01-HC-95165, N01-HC-95166, N01-HC-95167, N01-HC-95168, N01-HC-95169, UL1-TR-000040, UL1-TR-001079, UL1-TR-001420, UL1-TR-001881, and DK063491. Funding support for the neighborhood scales dataset was provided by R01HL071759. The MESA Epigenomics and Transcriptomics Studies were funded by NIH Grants 1R01HL101250, 1RF1AG054474, R01HL126477, R01DK101921, and R01HL135009. Analysis for this study was provided by R01HL141292.

## Conflict of Interest

The authors declare that the research was conducted in the absence of any commercial or financial relationships that could be construed as a potential conflict of interest.

## Publisher's Note

All claims expressed in this article are solely those of the authors and do not necessarily represent those of their affiliated organizations, or those of the publisher, the editors and the reviewers. Any product that may be evaluated in this article, or claim that may be made by its manufacturer, is not guaranteed or endorsed by the publisher.

## References

[1] McLaren L. Socioeconomic status and obesity. Epidemiol Rev. (2007) 29:29–48. 10.1093/epirev/mxm001 17478442

[2] Sobal J Stunkard AJ. Socioeconomic status and obesity: a review of the literature. Psychol Bull. (1989) 105:260–75. 10.1037/0033-2909.105.2.260 2648443

[3] Lee C Park S. Examining cumulative inequality in the association between childhood SES and BMI from midlife to old age. J Gerontol B Psychol Sci Soc Sci. (2020) 75:1264–74. 10.1093/geroni/igz038.1274 31168579 PMC7265812

[4] Kivimäki M Smith GD Elovainio M Pulkki L Keltikangas-Järvinen L Talttonen L. Socioeconomic circumstances in childhood and blood pressure in adulthood: the cardiovascular risk in young Finns study. Ann Epidemiol. (2006) 16:737–42. 10.1016/j.annepidem.2006.01.004 16843680

[5] Leng B Jin Y Li G Chen L Jin N. Socioeconomic status and hypertension: a meta-analysis. J Hypertens. (2015) 33:221–9. 10.1097/HJH.0000000000000428 25479029

[6] Matthews KA Kiefe CI Lewis CE Liu K Sidney S Yunis C. Socioeconomic trajectories and incident hypertension in a biracial cohort of young adults. Hypertension. (2002) 39:772–6. 10.1161/hy0302.105682 11897761

[7] Maty SC Everson-Rose SA Haan MN Raghunathan TE Kaplan GA. Education, income, occupation, and the 34-year incidence (1965-99) of Type 2 diabetes in the Alameda County Study. Int J Epidemiol. (2005) 34:1274–81. 10.1093/ije/dyi167 16120636 PMC3172611

[8] Tamayo T Christian H Rathmann W. Impact of early psychosocial factors (childhood socioeconomic factors and adversities) on future risk of type 2 diabetes, metabolic disturbances and obesity: a systematic review. BMC Public Health. (2010) 10:525. 10.1186/1471-2458-10-525 20809937 PMC2940917

[9] Gustafsson PE Janlert U Theorell T Westerlund H Hammarström A. Fetal and life course origins of serum lipids in mid-adulthood: results from a prospective cohort study. BMC Public Health. (2010) 10:484. 10.1186/1471-2458-10-484 20712860 PMC2936420

[10] Brunner E Shipley MJ Blane D Smith GD Marmot MG. When does cardiovascular risk start? Past and present socioeconomic circumstances and risk factors in adulthood. J Epidemiol Community Health. (1999) 53:757–64. 10.1136/jech.53.12.757 10656084 PMC1756821

[11] Brown H Becker F Antwi K. Association between lipid biomarkers, physical activity, and socioeconomic status in a population-based cross-sectional study in the UK. Sports Med Open. (2015) 2:25. 10.1186/s40798-016-0049-9 27366657 PMC4904024

[12] Kavanagh A Bentley RJ Turrell G Shaw J Dunstan D Subramanian SV. Socioeconomic position, gender, health behaviours and biomarkers of cardiovascular disease and diabetes. Soc Sci Med. (2010) 71:1150–60. 10.1016/j.socscimed.2010.05.038 20667641

[13] Pollitt RA Rose KM Kaufman JS. Evaluating the evidence for models of life course socioeconomic factors and cardiovascular outcomes: a systematic review. BMC Public Health. (2005) 5:7. 10.1186/1471-2458-5-7 15661071 PMC548689

[14] Diez Roux AV Merkin SS Arnett D Chambless L Massing M Nieto FJ. Neighborhood of residence and incidence of coronary heart disease. N Engl J Med. (2001) 345:99–106. 10.1056/NEJM200107123450205 11450679

[15] Sundquist K Winkleby M Ahlén H Johansson SE. Neighborhood socioeconomic environment and incidence of coronary heart disease: a follow-up study of 25,319 women and men in Sweden. Am J Epidemiol. (2004) 159:655–62. 10.1093/aje/kwh096 15033643

[16] Janssen I Boyce WF Simpson K Pickett W. Influence of individual- and area-level measures of socioeconomic status on obesity, unhealthy eating, and physical inactivity in Canadian adolescents. Am J Clin Nutr. (2006) 83:139–45. 10.1093/ajcn/83.1.139 16400062

[17] Kivimäki M Vahtera J Tabák AG Halonen JI Vineis P Pentti J. Neighbourhood socioeconomic disadvantage, risk factors, and diabetes from childhood to middle age in the Young Finns Study: a cohort study. Lancet Public Health. (2018) 3:e365–e73. 10.1016/S2468-2667(18)30111-7 30030110 PMC6079015

[18] Diez Roux AV Mair C. Neighborhoods and health. Ann N Y Acad Sci. (2010) 1186:125–45. 10.1111/j.1749-6632.2009.05333.x 20201871

[19] Mujahid MS Diez Roux AV Shen M Gowda D Sánchez B Shea S. Relation between neighborhood environments and obesity in the Multi-Ethnic Study of Atherosclerosis. Am J Epidemiol. (2008) 167:1349–57. 10.1093/aje/kwn047 18367469

[20] Kaiser P Diez Roux AV Mujahid M Carnethon M Bertoni A Adar SD. Neighborhood environments and incident hypertension in the multi-ethnic study of atherosclerosis. Am J Epidemiol. (2016) 183:988–97. 10.1093/aje/kwv296 27188946 PMC4887578

[21] Christine PJ Auchincloss AH Bertoni AG Carnethon MR Sánchez BN Moore K. Longitudinal associations between neighborhood physical and social environments and incident type 2 diabetes mellitus: the multi-ethnic study of atherosclerosis (MESA). JAMA Intern Med. (2015) 175:1311–20. 10.1001/jamainternmed.2015.2691 26121402 PMC4799846

[22] Unger E Diez-Roux AV Lloyd-Jones DM Mujahid MS Nettleton JA Bertoni A. Association of neighborhood characteristics with cardiovascular health in the multi-ethnic study of atherosclerosis. Circ Cardiovasc Qual Outcomes. (2014) 7:524–31. 10.1161/CIRCOUTCOMES.113.000698 25006187 PMC4172357

[23] Jaenisch R Bird A. Epigenetic regulation of gene expression: how the genome integrates intrinsic and environmental signals. Nat Genet. (2003) 33(Suppl):245–54. 10.1038/ng1089 12610534

[24] McGowan PO Szyf M. The epigenetics of social adversity in early life: implications for mental health outcomes. Neurobiol Dis. (2010) 39:66–72. 10.1016/j.nbd.2009.12.026 20053376

[25] Olden K Olden HA Lin YS. The role of the epigenome in translating neighborhood disadvantage into health disparities. Curr Environ Health Rep. (2015) 2:163–70. 10.1007/s40572-015-0048-x 26231365

[26] Giurgescu C Nowak AL Gillespie S Nolan TS Anderson CM Ford JL. Neighborhood environment and DNA methylation: implications for cardiovascular disease risk. J Urban Health. (2019) 96:23–34. 10.1007/s11524-018-00341-1 30635842 PMC6430282

[27] Notterman DA Mitchell C. Epigenetics and understanding the impact of social determinants of health. Pediatr Clin North Am. (2015) 62:1227–40. 10.1016/j.pcl.2015.05.012 26318949 PMC4555996

[28] Borghol N Suderman M McArdle W Racine A Hallett M Pembrey M. Associations with early-life socio-economic position in adult DNA methylation. Int J Epidemiol. (2012) 41:62–74. 10.1093/ije/dyr147 22422449 PMC3304522

[29] Needham BL Smith JA Zhao W Wang X Mukherjee B Kardia SL. Life course socioeconomic status and DNA methylation in genes related to stress reactivity and inflammation: the multi-ethnic study of atherosclerosis. Epigenetics. (2015) 10:958–69. 10.1080/15592294.2015.1085139 26295359 PMC4844216

[30] Tehranifar P Wu HC Fan X Flom JD Ferris JS Cho YH. Early life socioeconomic factors and genomic DNA methylation in mid-life. Epigenetics. (2013) 8:23–7. 10.4161/epi.22989 23196856 PMC3549876

[31] Bush NR Edgar RD Park M MacIsaac JL McEwen LM Adler NE. The biological embedding of early-life socioeconomic status and family adversity in children's genome-wide DNA methylation. Epigenomics. (2018) 10:1445–61. 10.2217/epi-2018-0042 30351206 PMC6462839

[32] Stringhini S Polidoro S Sacerdote C Kelly RS van Veldhoven K Agnoli C. Life-course socioeconomic status and DNA methylation of genes regulating inflammation. Int J Epidemiol. (2015) 44:1320–30. 10.1093/ije/dyv060 25889032

[33] Smith JA Zhao W Wang X Ratliff SM Mukherjee B Kardia SLR. Neighborhood characteristics influence DNA methylation of genes involved in stress response and inflammation: the multi-ethnic study of atherosclerosis. Epigenetics. (2017) 12:662–73. 10.1080/15592294.2017.1341026 28678593 PMC5687339

[34] Lei MK Beach SR Simons RL Philibert RA. Neighborhood crime and depressive symptoms among African American women: genetic moderation and epigenetic mediation of effects. Soc Sci Med. (2015) 146:120–8. 10.1016/j.socscimed.2015.10.035 26513121 PMC4655437

[35] Baccarelli A Wright R Bollati V Litonjua A Zanobetti A Tarantini L. Ischemic heart disease and stroke in relation to blood DNA methylation. Epidemiology. (2010) 21:819–28. 10.1097/EDE.0b013e3181f20457 20805753 PMC3690659

[36] Turunen MP Aavik E Ylä-Herttuala S. Epigenetics and atherosclerosis. Biochim Biophys Acta. (2009) 1790:886–91. 10.1016/j.bbagen.2009.02.008 19233248

[37] Smolarek I Wyszko E Barciszewska AM Nowak S Gawronska I Jablecka A. Global DNA methylation changes in blood of patients with essential hypertension. Med Sci Monit. (2010) 16:CR149–55. 20190686

[38] Fernández-Sanlés A Sayols-Baixeras S Subirana I Degano IR Elosua R. Association between DNA methylation and coronary heart disease or other atherosclerotic events: a systematic review. Atherosclerosis. (2017) 263:325–33. 10.1016/j.atherosclerosis.2017.05.022 28577936

[39] Talens RP Jukema JW Trompet S Kremer D Westendorp RG Lumey LH. Hypermethylation at loci sensitive to the prenatal environment is associated with increased incidence of myocardial infarction. Int J Epidemiol. (2012) 41:106–15. 10.1093/ije/dyr153 22101166 PMC3663184

[40] Sun YV Lazarus A Smith JA Chuang YH Zhao W Turner ST. Gene-specific DNA methylation association with serum levels of C-reactive protein in African Americans. PLoS ONE. (2013) 8:e73480. 10.1371/journal.pone.0073480 23977389 PMC3747126

[41] Westerman K Sebastiani P Jacques P Liu S DeMeo D Ordovás JM. methylation modules associate with incident cardiovascular disease and cumulative risk factor exposure. Clin Epigenetics. (2019) 11:142. 10.1186/s13148-019-0705-2 31615550 PMC6792327

[42] Agha G Mendelson MM Ward-Caviness CK Joehanes R Huan T Gondalia R. Blood leukocyte DNA methylation predicts risk of future myocardial infarction and coronary heart disease. Circulation. (2019) 140:645–57. 10.1161/CIRCULATIONAHA.118.039357 31424985 PMC6812683

[43] Shen Y Peng C Bai Q Ding Y Yi X Du H. Epigenome-wide association study indicates hypomethylation of MTRNR2L8 in large-artery atherosclerosis stroke. Stroke. (2019) 50:1330–8. 10.1161/STROKEAHA.118.023436 31084332

[44] Qin X Karlsson IK Wang Y Li X Pedersen N Reynolds CA. The epigenetic etiology of cardiovascular disease in a longitudinal Swedish twin study. Clin Epigenetics. (2021) 13:129. 10.1186/s13148-021-01113-6 34167563 PMC8223329

[45] Wahl S Drong A Lehne B Loh M Scott WR Kunze S. Epigenome-wide association study of body mass index, and the adverse outcomes of adiposity. Nature. (2017) 541:81–6. 10.1038/nature20784 28002404 PMC5570525

[46] Walaszczyk E Luijten M Spijkerman AMW Bonder MJ Lutgers HL Snieder H. DNA methylation markers associated with type 2 diabetes, fasting glucose and HbA. Diabetologia. (2018) 61:354–68. 10.1007/s00125-017-4497-7 29164275 PMC6448925

[47] Meeks KAC Henneman P Venema A Addo J Bahendeka S Burr T. Epigenome-wide association study in whole blood on type 2 diabetes among sub-Saharan African individuals: findings from the RODAM study. Int J Epidemiol. (2019) 48:58–70. 10.1093/ije/dyy171 30107520 PMC6380309

[48] Florath I Butterbach K Heiss J Bewerunge-Hudler M Zhang Y Schöttker B. Type 2 diabetes and leucocyte DNA methylation: an epigenome-wide association study in over 1,500 older adults. Diabetologia. (2016) 59:130–8. 10.1007/s00125-015-3773-7 26433941

[49] Richard MA Huan T Ligthart S Gondalia R Jhun MA Brody JA. DNA methylation analysis identifies loci for blood pressure regulation. Am J Hum Genet. (2017) 101:888–902. 10.1016/j.ajhg.2017.09.028 29198723 PMC5812919

[50] Jhun MA Mendelson M Wilson R Gondalia R Joehanes R Salfati E. A multi-ethnic epigenome-wide association study of leukocyte DNA methylation and blood lipids. Nat Commun. (2021) 12:3987. 10.1038/s41467-021-24600-z 34183656 PMC8238961

[51] Loucks EB Huang YT Agha G Chu S Eaton CB Gilman SE. Epigenetic mediators between childhood socioeconomic disadvantage and mid-life body mass index: the new england family study. Psychosom Med. (2016) 78:1053–65. 10.1097/PSY.0000000000000411 27768648 PMC7380568

[52] Chu SH Loucks EB Kelsey KT Gilman SE Agha G Eaton CB. Sex-specific epigenetic mediators between early life social disadvantage and adulthood BMI. Epigenomics. (2018) 10:707–22. 10.2217/epi-2017-0146 29888956 PMC6367732

[53] Bild DE Bluemke DA Burke GL Detrano R Diez Roux AV Folsom AR. Multi-ethnic study of atherosclerosis: objectives and design. Am J Epidemiol. (2002) 156:871–81. 10.1093/aje/kwf113 12397006

[54] Liu Y Ding J Reynolds LM Lohman K Register TC De La Fuente A. Methylomics of gene expression in human monocytes. Hum Mol Genet. (2013) 22:5065–74. 10.1093/hmg/ddt356 23900078 PMC3836482

[55] Du P Kibbe WA Lin SM. Lumi: a pipeline for processing Illumina microarray. Bioinformatics. (2008) 24:1547–8. 10.1093/bioinformatics/btn224 18467348

[56] Peters TJ Buckley MJ Statham AL Pidsley R Samaras KV Lord R. *De novo* identification of differentially methylated regions in the human genome. Epigenetics Chromatin. (2015) 8:6. 10.1186/1756-8935-8-6 25972926 PMC4429355

[57] Bibikova M Barnes B Tsan C Ho V Klotzle B Le JM. High density DNA methylation array with single CpG site resolution. Genomics. (2011) 98:288–95. 10.1016/j.ygeno.2011.07.007 21839163

[58] Desai S Alva S. Maternal education and child health: is there a strong causal relationship? Demography. (1998) 35:71–81. 10.2307/3004028 9512911

[59] Mujahid MS Diez Roux AV Morenoff JD Raghunathan T. Assessing the measurement properties of neighborhood scales: from psychometrics to ecometrics. Am J Epidemiol. (2007) 165:858–67. 10.1093/aje/kwm040 17329713

[60] Kim DS Li YK Bell GA Burt AA Vaisar T Hutchins PM. Concentration of smaller high-density lipoprotein particle (HDL-P) is inversely correlated with carotid intima media thickening after confounder adjustment: the multi ethnic study of atherosclerosis (MESA). J Am Heart Assoc. (2016) 5:977. 10.1161/JAHA.115.002977 27207961 PMC4889175

[61] Paramsothy P Knopp RH Bertoni AG Blumenthal RS Wasserman BA Tsai MY. Association of combinations of lipid parameters with carotid intima-media thickness and coronary artery calcium in the MESA (Multi-Ethnic Study of Atherosclerosis). J Am Coll Cardiol. (2010) 56:1034–41. 10.1016/j.jacc.2010.01.073 20846602

[62] Psaty BM Lee M Savage PJ Rutan GH German PS Lyles M. Assessing the use of medications in the elderly: methods and initial experience in the Cardiovascular Health Study. The Cardiovascular Health Study Collaborative Research Group. J Clin Epidemiol. (1992) 45:683–92. 10.1016/0895-4356(92)90143-B 1607909

[63] Smith NL Psaty BM Heckbert SR Tracy RP Cornell ES. The reliability of medication inventory methods compared to serum levels of cardiovascular drugs in the elderly. J Clin Epidemiol. (1999) 52:143–6. 10.1016/S0895-4356(98)00141-3 10201655

[64] Karlsson Linnér R Marioni RE Rietveld CA Simpkin AJ Davies NM Watanabe K. An epigenome-wide association study meta-analysis of educational attainment. Mol Psychiatry. (2017) 22:1680–90. 10.1038/mp.2017.210 29086770 PMC6372242

[65] Sun D Zhang T Su S Hao G Chen T Li QZ. Body mass index drives changes in DNA methylation: a longitudinal study. Circ Res. (2019) 125:824–33. 10.1161/CIRCRESAHA.119.315397 31510868 PMC6786955

[66] Huang Y-T. Genome-wide analyses of sparse mediation effects under composite null hypotheses. Ann Appl Stat. (2019) 13:60–84. 10.1214/18-AOAS1181

[67] Tingley D Yamamoto T Hirose K Keele L Imai K. Mediation: R package for causal mediation analysis. J Statist Software. (2014) 59:1–38. 10.18637/jss.v059.i05

[68] Zhang H Zheng Y Zhang Z Gao T Joyce B Yoon G. Estimating and testing high-dimensional mediation effects in epigenetic studies. Bioinformatics. (2016) 32:3150–4. 10.1093/bioinformatics/btw351 27357171 PMC5048064

[69] Zhang H Zheng Y Hou L Zheng C Liu L. Mediation analysis for survival data with High-Dimensional mediators. Bioinformatics. (2021) 37:3815–21. 10.1093/bioinformatics/btab564 34343267 PMC8570823

[70] Ogden CL Lamb MM Carroll MD Flegal KM. Obesity and Socioeconomic Status in Adults: United States, 2005-2008. NCHS Data Brief No 50. Hyattsville, MD: National Center for Health Statistics (2010). 21211165

[71] Bann D Johnson W Li L Kuh D Hardy R. Socioeconomic inequalities in body mass index across adulthood: coordinated analyses of individual participant data from three British Birth Cohort Studies Initiated in 1946, 1958 and 1970. PLoS Med. (2017) 14:e1002214. 10.1371/journal.pmed.1002214 28072856 PMC5224787

[72] Hajizadeh M Campbell MK Sarma S. Socioeconomic inequalities in adult obesity risk in Canada: trends and decomposition analyses. Eur J Health Econ. (2014) 15:203–21. 10.1007/s10198-013-0469-0 23543117

[73] Vernay M Malon A Oleko A Salanave B Roudier C Szego E. Association of socioeconomic status with overall overweight and central obesity in men and women: the French Nutrition and Health Survey 2006. BMC Public Health. (2009) 9:215. 10.1186/1471-2458-9-215 19573222 PMC2714511

[74] Huan T Joehanes R Song C Peng F Guo Y Mendelson M. Genome-wide identification of DNA methylation QTLs in whole blood highlights pathways for cardiovascular disease. Nat Commun. (2019) 10:4267. 10.1038/s41467-019-12228-z 31537805 PMC6753136

[75] Phipson B Maksimovic J Oshlack A. MissMethyl: an R package for analyzing data from Illumina's HumanMethylation450 platform. Bioinformatics. (2016) 32:286–8. 10.1093/bioinformatics/btv560 26424855

[76] Yu G Wang LG Han Y He QY. ClusterProfiler: an R package for comparing biological themes among gene clusters. OMICS. (2012) 16:284–7. 10.1089/omi.2011.0118 22455463 PMC3339379

[77] Kundaje A Meuleman W Ernst J Bilenky M Yen A Heravi-Moussavi A. Integrative analysis of 111 reference human epigenomes. Nature. (2015) 518:317–30. 10.1038/nature14248 25693563 PMC4530010

[78] Watanabe K Matsumoto A Tsuda H Iwamoto S. N4BP2L1 interacts with dynactin and contributes to GLUT4 trafficking and glucose uptake in adipocytes. J Diabetes Investig. (2021) 12:1958–66. 10.1111/jdi.13623 34197691 PMC8565410

[79] Geurts YM Dugué PA Joo JE Makalic E Jung CH Guan W. Novel associations between blood DNA methylation and body mass index in middle-aged and older adults. Int J Obes. (2018) 42:887–96. 10.1038/ijo.2017.269 29278407

[80] Gomes AV. Genetics of proteasome diseases. Scientifica. (2013) 2013:637629. 10.1155/2013/637629 24490108 PMC3892944

[81] Alsmadi O Muiya P Khalak H Al-Saud H Meyer BF Al-Mohanna F. Haplotypes encompassing the KIAA0391 and PSMA6 gene cluster confer a genetic link for myocardial infarction and coronary artery disease. Ann Hum Genet. (2009) 73:475–83. 10.1111/j.1469-1809.2009.00534.x 19624571

[82] Batista IAA Helguero LA. Biological processes and signal transduction pathways regulated by the protein methyltransferase SETD7 and their significance in cancer. Signal Transduct Target Ther. (2018) 3:19. 10.1038/s41392-018-0017-6 30013796 PMC6043541

[83] Wunderlich CM Hövelmeyer N Wunderlich FT. Mechanisms of chronic JAK-STAT3-SOCS3 signaling in obesity. JAKSTAT. (2013) 2:e23878. 10.4161/jkst.23878 24058813 PMC3710326

[84] Xu K Zhang X Wang Z Hu Y Sinha R. Epigenome-wide association analysis revealed that SOCS3 methylation influences the effect of cumulative stress on obesity. Biol Psychol. (2018) 131:63–71. 10.1016/j.biopsycho.2016.11.001 27826092 PMC5419875

[85] Mizuarai S Miki S Araki H Takahashi K Kotani H. Identification of dicarboxylate carrier Slc25a10 as malate transporter in *de novo* fatty acid synthesis. J Biol Chem. (2005) 280:32434–41. 10.1074/jbc.M503152200 16027120

[86] Li C Li L Yang M Zeng L Sun L. PACS-2: a key regulator of mitochondria-associated membranes (MAMs). Pharmacol Res. (2020) 160:105080. 10.1016/j.phrs.2020.105080 32673704

[87] Hellmann J Sansbury BE Holden CR Tang Y Wong B Wysoczynski M. CCR7 maintains nonresolving lymph node and adipose inflammation in obesity. Diabetes. (2016) 65:2268–81. 10.2337/db15-1689 27207557 PMC4955992

[88] Yang X Yue Y Xiong S. Dpep2 emerging as a modulator of macrophage inflammation confers protection against CVB3-induced viral myocarditis. Front Cell Infect Microbiol. (2019) 9:57. 10.3389/fcimb.2019.00057 30899700 PMC6416667

[89] Heemskerk MM Giera M Bouazzaoui FE Lips MA Pijl H van Dijk KW. Increased PUFA content and 5-lipoxygenase pathway expression are associated with subcutaneous adipose tissue inflammation in obese women with type 2 diabetes. Nutrients. (2015) 7:7676–90. 10.3390/nu7095362 26378572 PMC4586557

[90] Pisano E Pacifico L Perla FM Liuzzo G Chiesa C Lavorato M. Upregulated monocyte expression of PLIN2 is associated with early arterial injury in children with overweight/obesity. Atherosclerosis. (2021) 327:68–75. 10.1016/j.atherosclerosis.2021.04.016 34044206

[91] Andrade S Morais T Sandovici I Seabra AL Constância M Monteiro MP. Adipose tissue epigenetic profile in obesity-related dysglycemia - a systematic review. Front Endocrinol. (2021) 12:681649. 10.3389/fendo.2021.681649 34290669 PMC8288106

[92] Sell H Habich C Eckel J. Adaptive immunity in obesity and insulin resistance. Nat Rev Endocrinol. (2012) 8:709–16. 10.1038/nrendo.2012.114 22847239

[93] McLaughlin T Ackerman SE Shen L Engleman E. Role of innate and adaptive immunity in obesity-associated metabolic disease. J Clin Invest. (2017) 127:5–13. 10.1172/JCI88876 28045397 PMC5199693

[94] Deng T Lyon CJ Minze LJ Lin J Zou J Liu JZ. Class II major histocompatibility complex plays an essential role in obesity-induced adipose inflammation. Cell Metab. (2013) 17:411–22. 10.1016/j.cmet.2013.02.009 23473035 PMC3619392

[95] Klimcáková E Roussel B Márquez-Quiñones A Kovácová Z Kováciková M Combes M. Worsening of obesity and metabolic status yields similar molecular adaptations in human subcutaneous and visceral adipose tissue: decreased metabolism and increased immune response. J Clin Endocrinol Metab. (2011) 96:E73–82. 10.1210/jc.2010-1575 21047918

[96] Shaikh SR Mitchell D Carroll E Li M Schneck J Edidin M. Differential effects of a saturated and a monounsaturated fatty acid on MHC class I antigen presentation. Scand J Immunol. (2008) 68:30–42. 10.1111/j.1365-3083.2008.02113.x 18533931 PMC2805012

[97] Sayols-Baixeras S Subirana I Lluis-Ganella C Civeira F Roquer J Do AN. Identification and validation of seven new loci showing differential DNA methylation related to serum lipid profile: an epigenome-wide approach. The REGICOR study. Hum Mol Genet. (2016) 25:4556–65. 10.1093/hmg/ddw285 28173150 PMC6284258

[98] Ligthart S Marzi C Aslibekyan S Mendelson MM Conneely KN Tanaka T. DNA methylation signatures of chronic low-grade inflammation are associated with complex diseases. Genome Biol. (2016) 17:255. 10.1186/s13059-016-1119-5 27955697 PMC5151130

[99] Bodhini D Gaal S Shatwan I Ramya K Ellahi B Surendran S. Interaction between TCF7L2 polymorphism and dietary fat intake on high density lipoprotein cholesterol. PLoS ONE. (2017) 12:e0188382. 10.1371/journal.pone.0188382 29182660 PMC5705148

[100] Yamada Y Kato K Oguri M Horibe H Fujimaki T Yasukochi Y. Identification of 12 novel loci that confer susceptibility to early-onset dyslipidemia. Int J Mol Med. (2019) 43:57–82. 10.3892/ijmm.2018.3943 30365130 PMC6257857

[101] Agmon E Stockwell BR. Lipid homeostasis and regulated cell death. Curr Opin Chem Biol. (2017) 39:83–9. 10.1016/j.cbpa.2017.06.002 28645028 PMC5581689

[102] Chang HT Liu CC Chen ST Yap YV Chang NS Sze CI. domain-containing oxidoreductase in neuronal injury and neurological diseases. Oncotarget. (2014) 5:11792–9. 10.18632/oncotarget.2961 25537520 PMC4322972

[103] Colin S Fanchon M Belloy L Bochem AE Copin C Derudas B. HDL does not influence the polarization of human monocytes toward an alternative phenotype. Int J Cardiol. (2014) 172:179–84. 10.1016/j.ijcard.2013.12.168 24456889

[104] Nofer JR Kehrel B Fobker M Levkau B Assmann G von Eckardstein A. and arteriosclerosis: beyond reverse cholesterol transport. Atherosclerosis. (2002) 161:1–16. 10.1016/S0021-9150(01)00651-7 11882312

[105] Yu XH Fu YC Zhang DW Yin K Tang CK. Foam cells in atherosclerosis. Clin Chim Acta. (2013) 424:245–52. 10.1016/j.cca.2013.06.006 23782937

[106] Dekkers KF Neele AE Jukema JW Heijmans BT de Winther MPJ. Human monocyte-to-macrophage differentiation involves highly localized gainand loss of DNA methylation at transcription factor binding sites. Epigenetics Chromatin. (2019) 12:34. 10.1186/s13072-019-0279-4 31171035 PMC6551876

[107] Lawrence T Natoli G. Transcriptional regulation of macrophage polarization: enabling diversity with identity. Nat Rev Immunol. (2011) 11:750–61. 10.1038/nri3088 22025054

[108] Zeng P Shao Z Zhou X. Statistical methods for mediation analysis in the era of high-throughput genomics: current successes and future challenges. Comput Struct Biotechnol J. (2021) 19:3209–24. 10.1016/j.csbj.2021.05.042 34141140 PMC8187160

[109] Liu Z Shen J Barfield R Schwartz J Baccarelli AA Lin X. Large-scale hypothesis testing for causal mediation effects with applications in genome-wide epigenetic studies. J Am Stat Assoc. (2021) 117:1–15. 10.1101/2020.09.20.20198226 PMC9385159 35989709

[110] Huang YT Chu S Loucks EB Lin CL Eaton CB Buka SL. Epigenome-wide profiling of DNA methylation in paired samples of adipose tissue and blood. Epigenetics. (2016) 11:227–36. 10.1080/15592294.2016.1146853 26891033 PMC4854552

[111] Winkleby MA Jatulis DE Frank E Fortmann SP. Socioeconomic status and health: how education, income, and occupation contribute to risk factors for cardiovascular disease. Am J Public Health. (1992) 82:816–20. 10.2105/AJPH.82.6.816 1585961 PMC1694190

[112] Metcalf P Scragg R Davis P. Relationship of different measures of socioeconomic status with cardiovascular disease risk factors and lifestyle in a New Zealand workforce survey. N Z Med J. (2007) 120:U2392. 17277809

[113] Caldwell JC. Education as a factor in mortality decline an examination of Nigerian data. Popul Stud. (1979) 33:395–413. 10.2307/217388819087334

[114] Aslam M Kingdon GG. Parental education and child health—understanding the pathways of impact in Pakistan. World Dev. (2012) 40:2014–32. 10.1016/j.worlddev.2012.05.007

[115] VanderWeele T. Explanation in Causal Inference: Methods for Mediation and Interaction. New York, NY: Oxford University Press (2015).

[116] Mathieu JE Taylor SR. Clarifying conditions and decision points for mediational type inferences in organizational behavior. J Org Behav. (2006) 27:1031–56. 10.1002/job.406

[117] Song Y Zhou X Zhang M Zhao W Liu Y Kardia SLR. Bayesian shrinkage estimation of high dimensional causal mediation effects in omics studies. Biometrics. (2019). 10.1101/467399 31733066 PMC7228845

[118] Song Y Zhou X Kang J Aung MT Zhang M Zhao W. Bayesian hierarchical models for high-dimensional mediation analysis with coordinated selection of correlated mediators. Stat Med. (2021) 40:6038–56. 10.1002/sim.9168 34404112 PMC9257993

[119] Song Y Zhou X Kang J Aung MT Zhang M Zhao W. Bayesian sparse mediation analysis with targeted penalization of natural indirect effects. J R Stat Soc Ser C Appl Stat. (2021) 70:1391–3912 10.1111/rssc.12518 34887595 PMC8653861

