# Supplementary material for: DNA Methylation Mediates the Association Between Individual and Neighborhood Social Disadvantage and Cardiovascular Risk Factors
Source: Front Cardiovasc Med. 2022 May 19;9:848768. doi: 10.3389/fcvm.2022.848768 (PMC9162507; doi:10.3389/fcvm.2022.848768)
Supplement: Supplementary file 1 [file Data_Sheet_1.docx]

Supplementary Material

# Supplementary Methods: Construction of Neighborhood Scores

Measures of neighborhood socioeconomic disadvantage and neighborhood social environment were constructed as described below. In order to assess the effects of long-term exposure to neighborhood characteristics, we used the weighted cumulative average of each of the neighborhood measures across all available MESA examinations (maximum of 5 exams, spanning 10 years from baseline to Exam 5), with the weights being proportional to the number of months the respondents resided in each neighborhood.

### 1.1 Neighborhood Socioeconomic Disadvantage

Neighborhood level scales for characteristics of socioeconomic status (SES) were obtained from the U.S. Census 2000 Summary File 1 and Summary File 3, American Community Survey (ACS) 2005-2009, and ACS 2007-2011 estimates at the census tract level. We conducted principal factor analysis with orthogonal rotation of 16 census variables which reflect aspects of crowding (percent of households with crowing greater than 1 person per room), education (percent of adults age 25 or older with at least a high school education and percent of adults age 25 or older with at least a Bachelor’s degree), occupation (percent of persons age 16 and older with executive, managerial, or professional occupation), income and wealth (median value of housing units, percent of housing units without a telephone, percent of housing units without a vehicle, median household income, percent of households with income of at least $50,000, percent of household with interest, dividend, or net rental income, and percent of household receiving public assistance), poverty (percent below poverty level), employment (percent of those age 16 or older who are unemployed and percent of those age 16 and older who are not in the labor force), and housing (percent of occupied housing units, percent of housing units that are owner occupied, and percent of persons living in same house as previous census). Variables that represent a better SES environment were reverse coded. Four factors were kept which reflects 73% of the variance explained. Weighted scales were created by multiplying the factor weights by the standardized variables, and increasing scores represents socioeconomic disadvantage. The first factor, which we used in these analyses, represents education, occupation, and income and wealth, and was highly weighted on % bachelor degree, % managerial occupation, median home value, % HS education, % interest/dividend/rental income, median household income, and % household income >$50,000. The scales are linked to MESA participants by census tract using Census 2000 data for years 2000-2004, ACS 2005- 2009 data for years 2005-2007, and ACS 2007-2011 data for years 2008-2012.

### 1.2 Neighborhood Social Environment

For the survey scales, information on neighborhood level characteristics was ascertained via questionnaire asking participants to rate the area within approximately 1 mile around their home collected in 2003-2005 and 2010-2012. Information was obtained from questionnaires administered to MESA participants and to a random auxiliary sample of other neighborhood residents in the New York, Baltimore, and Forsyth County study sites in 2003-2005 and all study sites in 2010-2012. Three neighborhood dimensions were assessed: aesthetic quality (3 items, “There is a lot of trash and litter on the street in my neighborhood”, “There is a lot of noise in my neighborhood”, and “My neighborhood is attractive”), safety (2 items, “I feel safe walking in my neighborhood day or night” and “Violence is a problem in my neighborhood”), and social cohesion (4 items, “People around here are willing to help their neighbors”, “People in my neighborhood generally get along with each other”, “People in my neighborhood can be trusted”, and “People in my neighborhood share the same values”). Responses for each item ranged from 1 (strongly agree) to 5 (strongly disagree). Questions were reverse coded when needed to indicate a higher score being a more positive or favorable environment. Scales were based on previous work and have acceptable internal consistency (Cronbach alpha 0.64-0.82).

Conditional empirical Bayes (CEB) estimates were derived from three level hierarchical linear models using HLM version 7.0 (Mujahid 2007). This method accounts for the nested structure of the data (i.e. scale items nested within individuals nested within neighborhoods). The estimates were conditioned on respondents’ gender, age, whether in MESA or CS sample, and study site. These estimates are a weighted average of the mean across census tracts where the weights are proportional to the reliability. The greater the variance across neighborhoods and the greater the sample size within a census tract, the greater the reliability. The measure has less reliability for a census tract if there is poor agreement between members of the census tract or there is a small sample size. The census tracts that have high reliability will have less shrinkage while census tracts with poor reliability will have greater shrinkage to the overall mean. The advantage to these estimates is that there is borrowing of information from other census tracts to improve the estimate for the unreliable tracts.

A summary social environment scale was calculated by summing the standardized CEB estimates for the three scales. The scales are linked to MESA participants by census tract using the survey closest in time to the exam date.

# Supplementary Figures


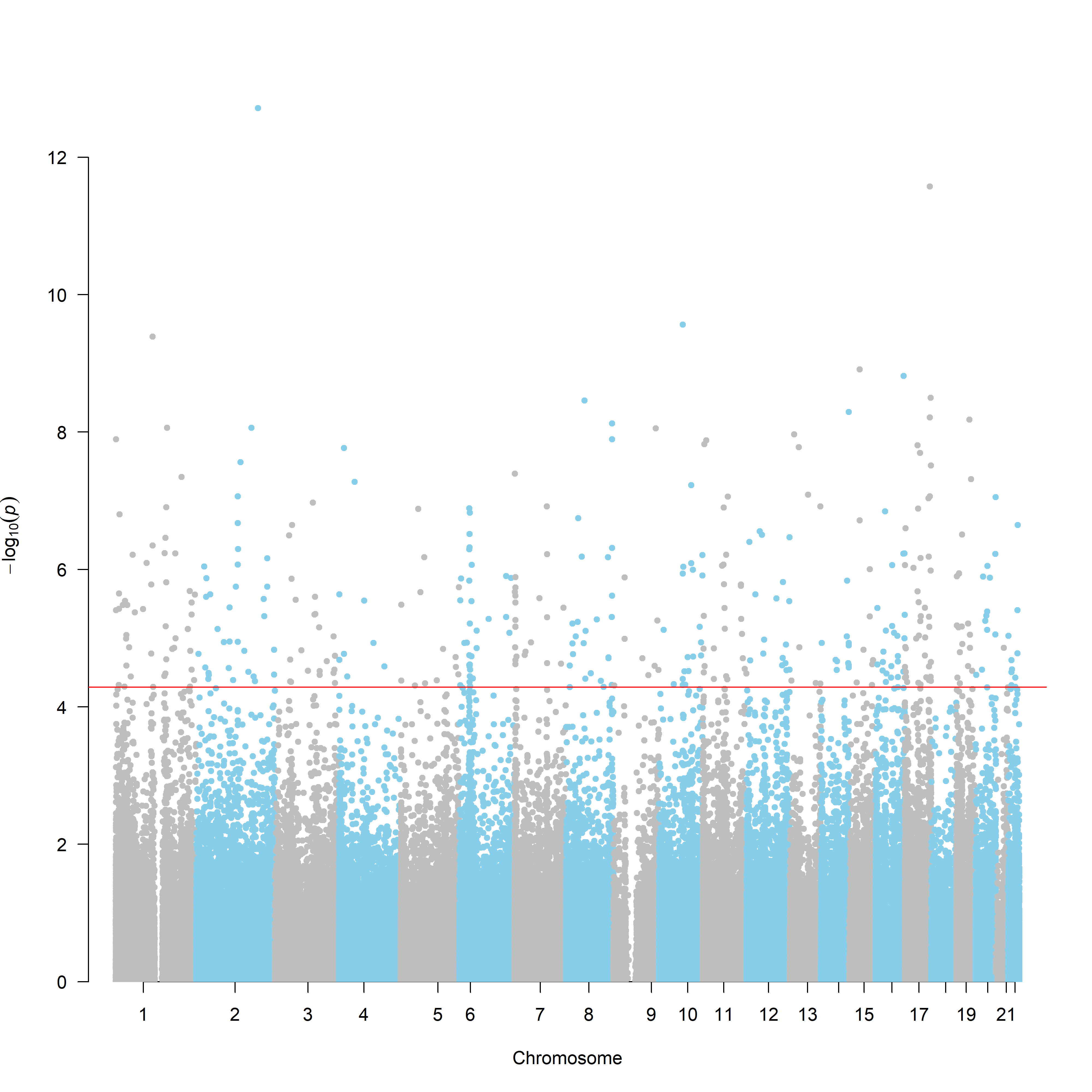


**Supplementary Figure 1**. Manhattan plot of the epigenetic mediation effects for the association between adult SES and BMI. The –log (p value) (Y axis) was plotted against the chromosomal position (GRCh37, x axis) for each CpG site. The red line indicates significance at FDR q<0.05.


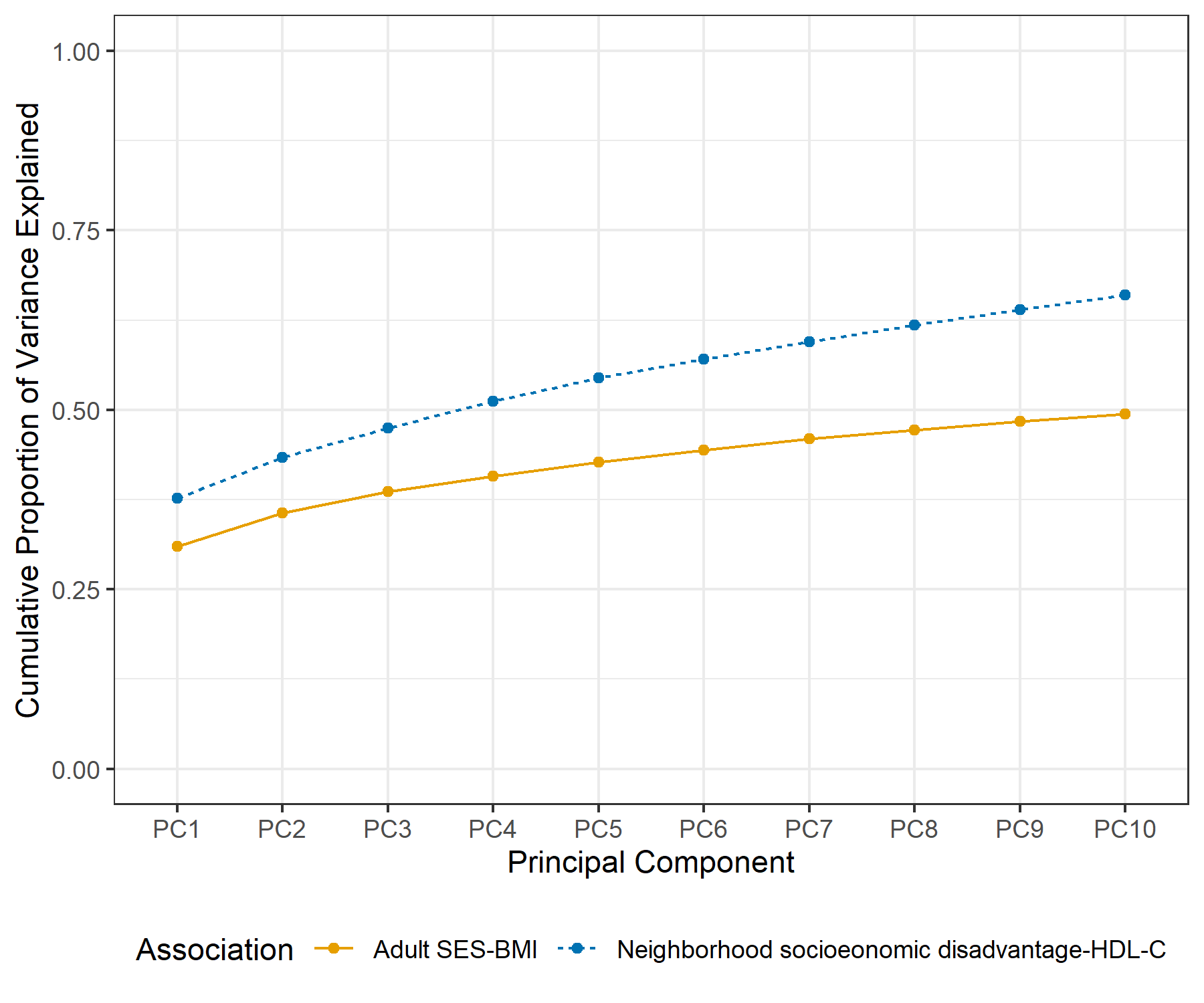


**Supplementary Figure 2**. Principal component (PC) analysis of mediating CpG sites. The cumulative proportion of variance explained by the first 10 PCs for the 410 CpG sites mediating the association between adult SES and BMI (solid yellow line) and the 43 CpG sites mediating the association between neighborhood socioeconomic disadvantage and HDL-C (dashed blue line) is provided for each PC.


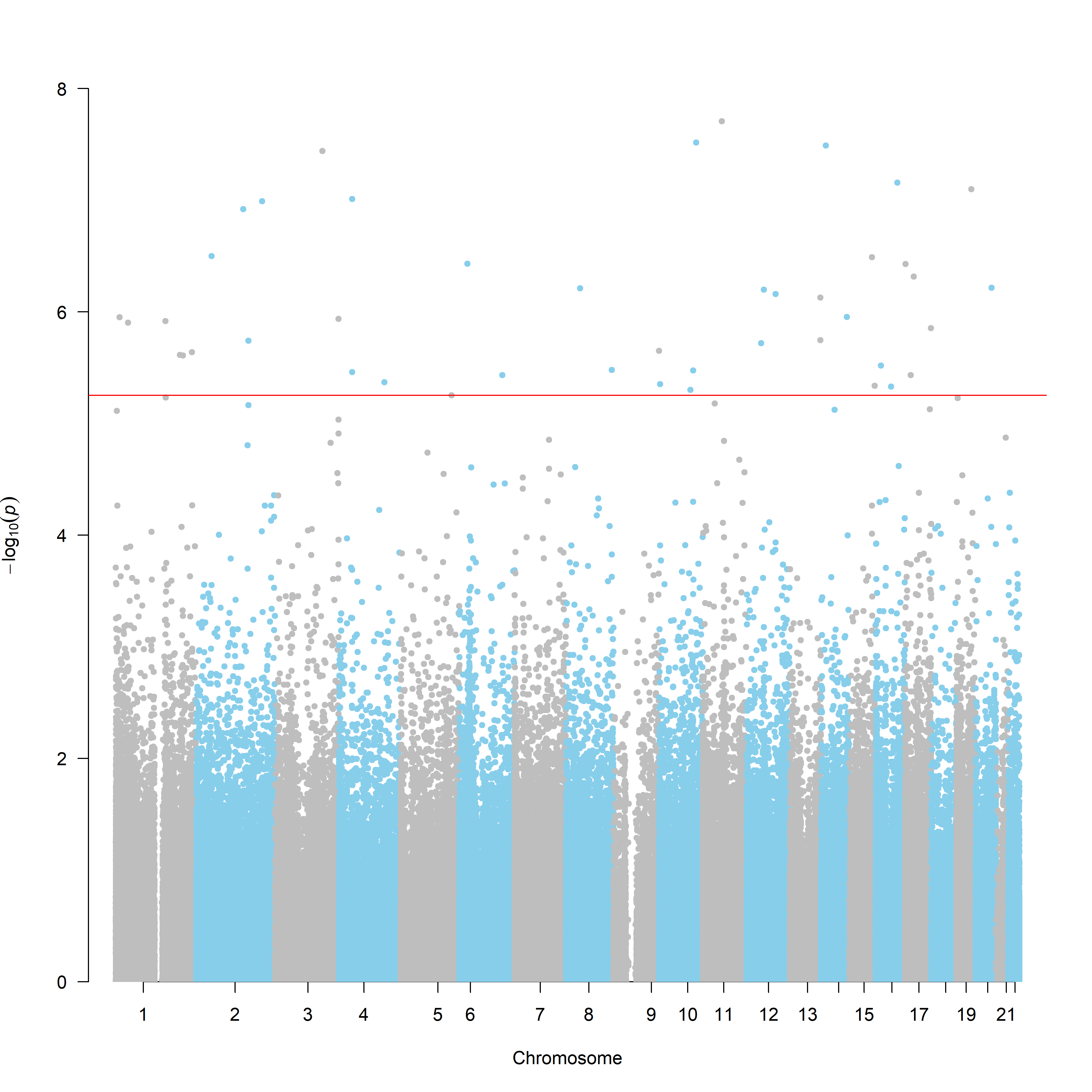


**Supplementary Figure 3**. Manhattan plot of the epigenetic mediation effects for the association between neighborhood socioeconomic disadvantage and HDL cholesterol. The –log (p value) (Y axis) was plotted against the chromosomal position (GRCh37, x axis) for each CpG site. The red line indicates significance at FDR q<0.05.


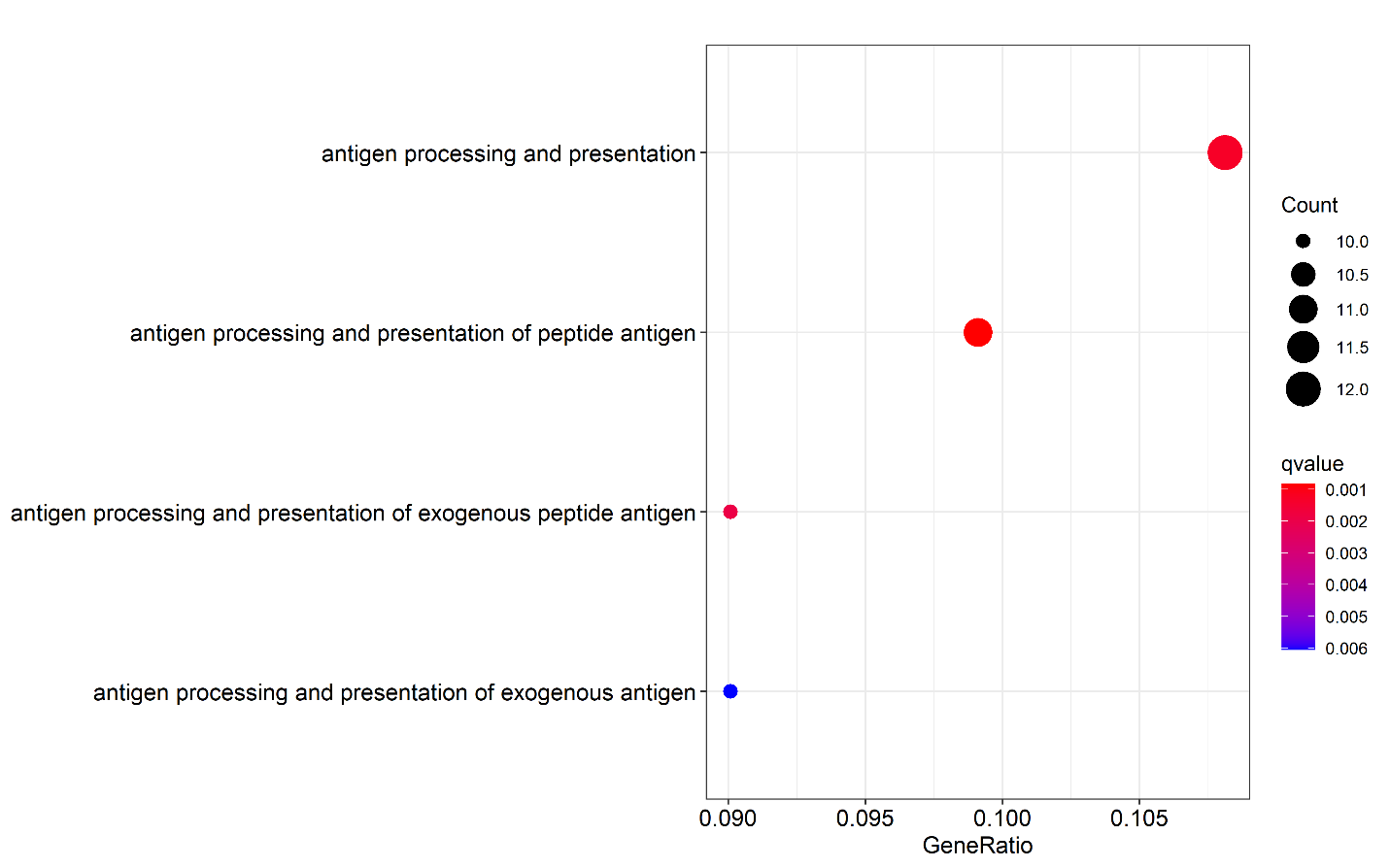


**Supplementary Figure 4**. Dotplot showing the results of the Gene Ontology (GO) biological processes over-representation analysis for the genes whose expression was associated with CpG sites mediating the relationship between adult SES and BMI. Dot size indicates number of genes in each GO category. Dot color indicates FDR q-value.


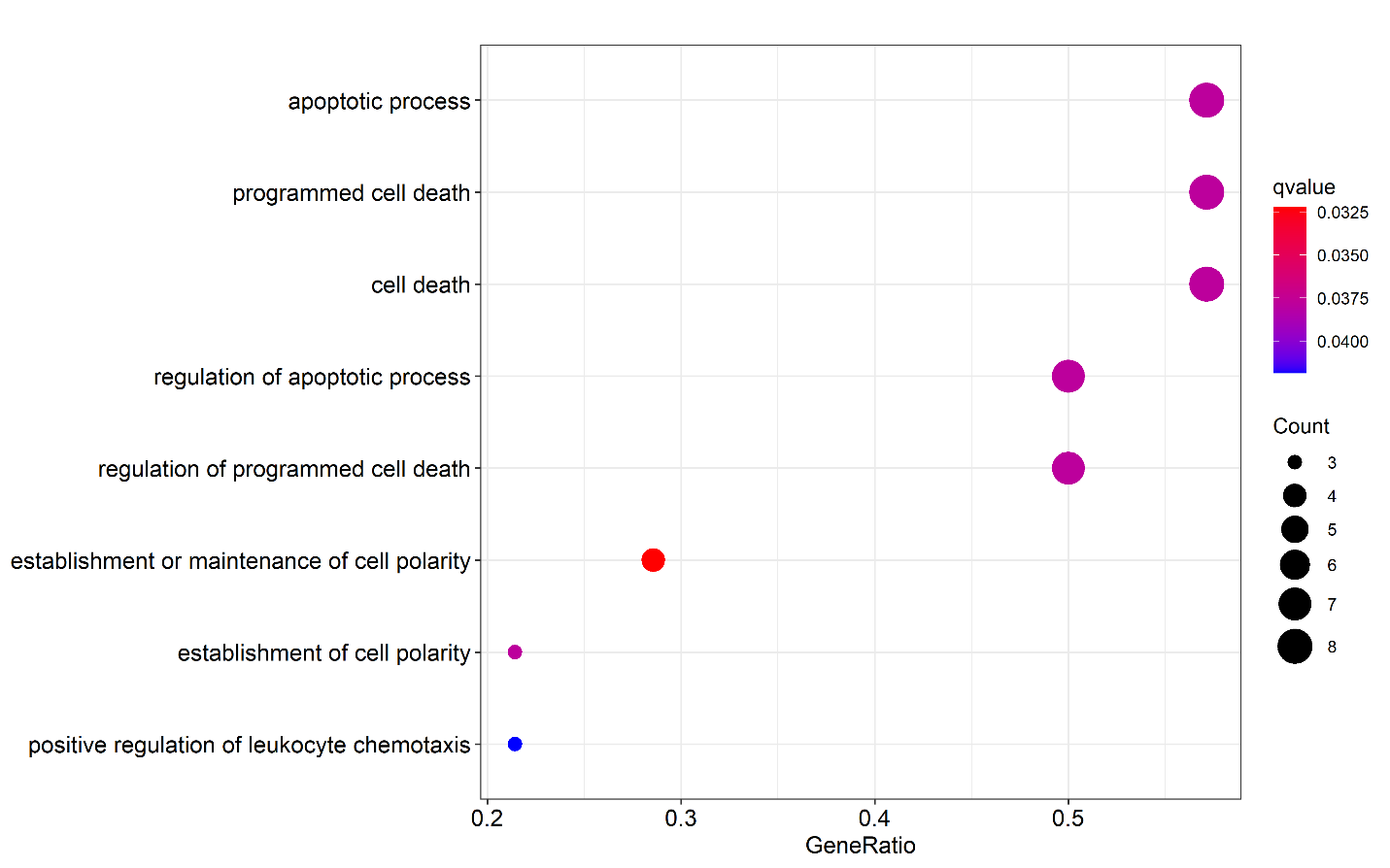


**Supplementary Figure 5**. Dotplot showing the results of the Gene Ontology (GO) biological processes over-representation analysis for the genes whose expression was associated with CpG sites mediating the relationship between neighborhood socioeconomic disadvantage and HDL-C. Dot size indicates number of genes in each GO category. Dot color indicates FDR q-value.


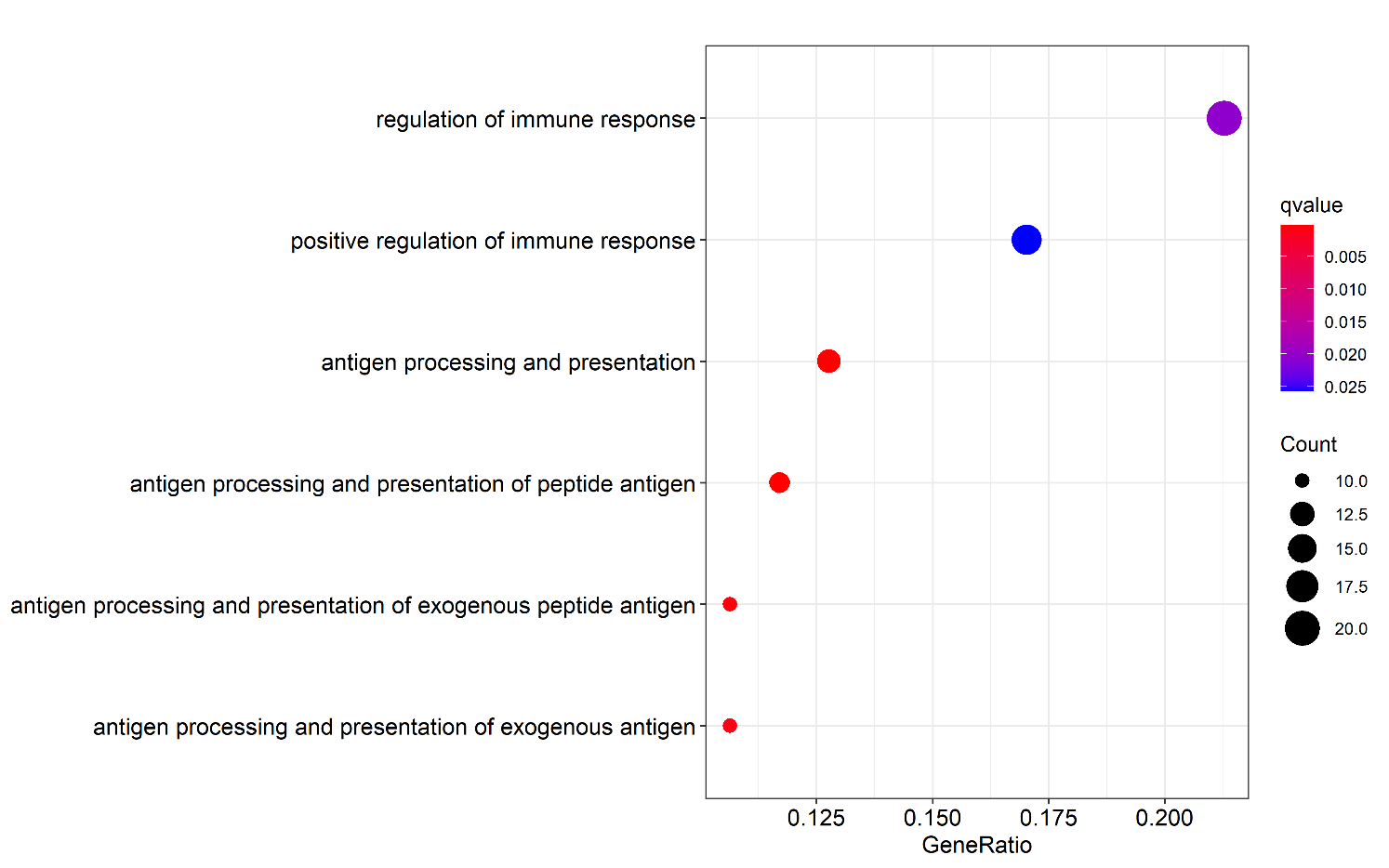


**Supplementary Figure 6.** Dotplot showing the results of the Gene Ontology (GO) biological processes over-representation analysis for the genes whose expression was associated with CpG mediators of the relationship between adult SES and BMI and in an active chromatin state in primary mononuclear cells. Dot size indicates number of genes in each GO category. Dot color indicates FDR q-value.


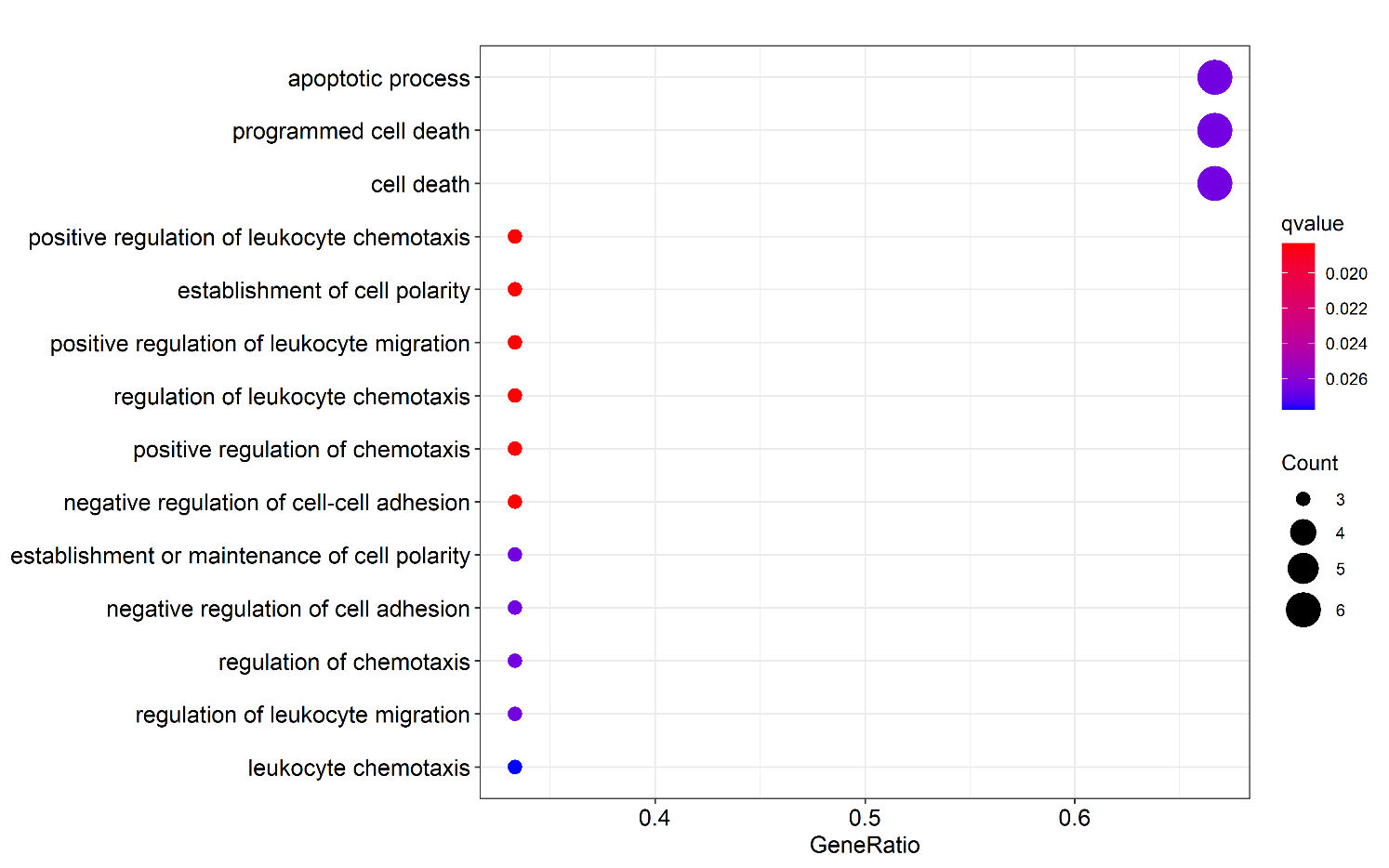


**Supplementary Figure 7**. Dotplot showing the results of the Gene Ontology (GO) biological processes over-representation analysis for the genes whose expression was associated with CpG sites mediating the relationship between neighborhood socioeconomic disadvantage and HDL-C and in an active chromatin state in primary mononuclear cells. Dot size indicates number of genes in each GO category. Dot color indicates FDR q-value.
